# A Highly Conserved Peptide Vaccine Candidate Activates Both Humoral and Cellular Immunity Against SARS-CoV-2 Variant Strains

**DOI:** 10.3389/fimmu.2021.789905

**Published:** 2021-12-07

**Authors:** Fengxia Gao, Jingjing Huang, Tingting Li, Chao Hu, Meiying Shen, Song Mu, Feiyang Luo, Shuyi Song, Yanan Hao, Wang Wang, Xiaojian Han, Chen Qian, Yingming Wang, Ruixin Wu, Luo Li, Shenglong Li, Aishun Jin

**Affiliations:** ^1^ Department of Immunology, College of Basic Medicine, Chongqing Medical University, Chongqing, China; ^2^ Chongqing Key Laboratory of Basic and Translational Research of Tumor Immunology, Chongqing Medical University, Chongqing, China; ^3^ Department of Endocrine Breast Surgery, The First Affiliated Hospital of Chongqing Medical University, Chongqing, China

**Keywords:** SARS-CoV-2 variants, RBD9.1, vaccine, humoral immune response, cellular immune response, immunological memory

## Abstract

Facing the imminent need for vaccine candidates with cross-protection against globally circulating severe acute respiratory syndrome coronavirus 2 (SARS-CoV-2) mutants, we present a conserved antigenic peptide RBD9.1 with both T-cell and B-cell epitopes. RBD9.1 can be recognized by coronavirus disease 2019 (COVID-19) convalescent serum, particularly for those with high neutralizing potency. Immunization with RBD9.1 can successfully induce the production of the receptor-binding domain (RBD)-specific antibodies in Balb/c mice. Importantly, the immunized sera exhibit sustained neutralizing efficacy against multiple dominant SARS-CoV-2 variant strains, including B.1.617.2 that carries a point mutation (S^L452R^) within the sequence of RBD9.1. Specifically, S^Y451^ and S^Y454^ are identified as the key amino acids for the binding of the induced RBD-specific antibodies to RBD9.1. Furthermore, we have confirmed that the RBD9.1 antigenic peptide can induce a S^448-456^ (NYNYLYRLF)-specific CD8^+^ T-cell response. Both RBD9.1-specific B cells and the S^448-456^-specific T cells can still be activated more than 3 months post the last immunization. This study provides a potential vaccine candidate that can generate long-term protective efficacy over SARS-CoV-2 variants, with the unique functional mechanism of activating both humoral and cellular immunity.

**Graphical Abstract:**
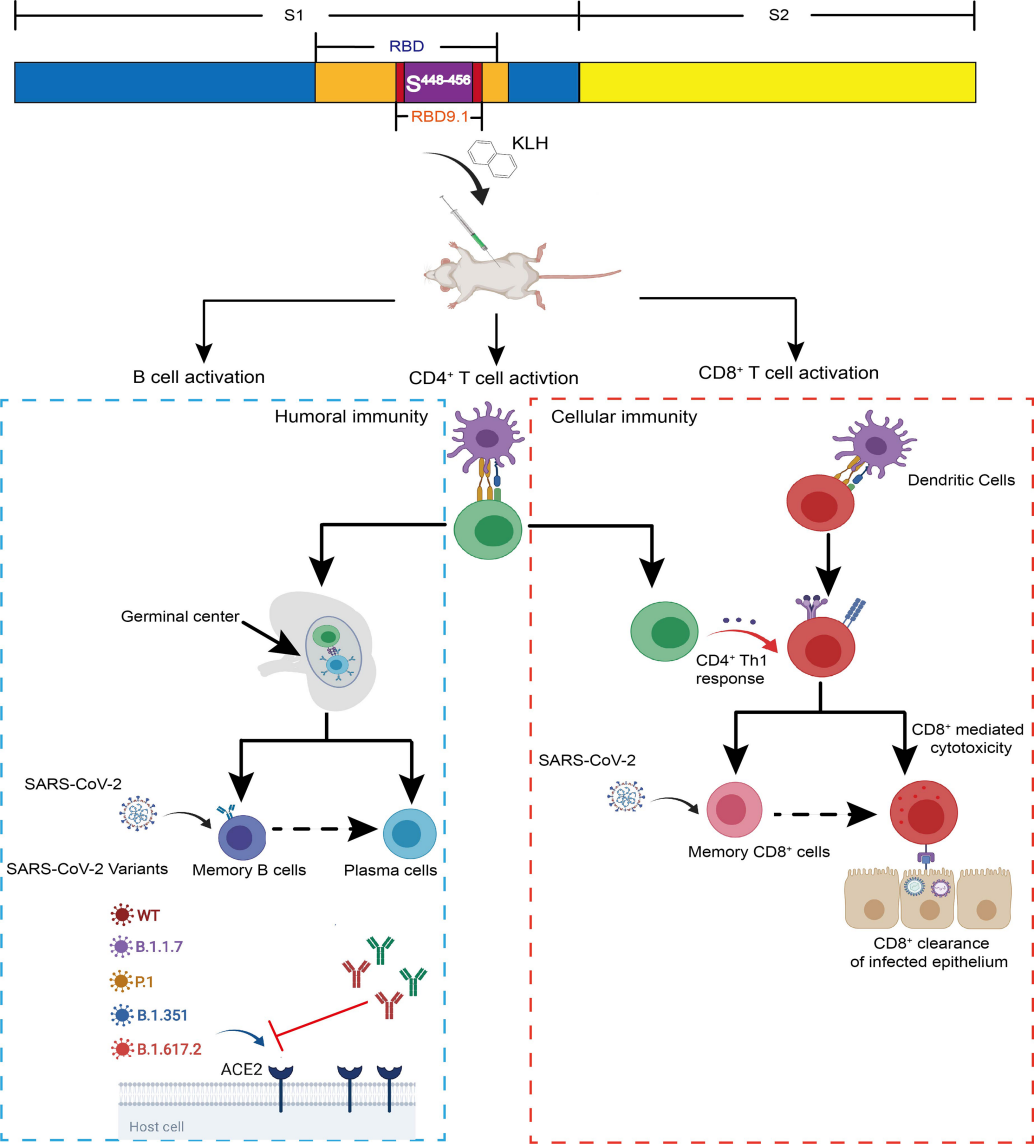


## Introduction

Vaccines against severe acute respiratory syndrome coronavirus 2 (SARS-CoV-2) may be the most effective way to improve the current social distancing resulting from protective measures and to recover from economic damage (1). With the acceleration of clinical trials, many countries have deployed national SARS-CoV-2 vaccination plans. However, the global emergence of multiple variant strains with enhanced infectivity over the last few months has presented great challenges for current vaccines (1–10).

So far, available coronavirus disease 2019 (COVID-19) vaccines include recombinant proteins, RNA vectors, virus-like particles (VLPs), and inactivated virus. All of these share the neutralizing mechanism to produce antibody responses against the wild-type Spike (S) protein of SARS-CoV-2 (11–15). It has been reported that vaccine candidates designed to target the receptor-binding domain (RBD) within the S protein are preferentially effective because of the competitive binding of induced antibodies in the serum to RBD (16, 17). Consequently, mutations in the RBD, such as S^E484K/Q^ shared by P.1, B.1.351, B.1.617.1, and B.1.617.2, or S^L452R^ presented in B.1.617.1 and B.1.617.2, may cause allosteric alterations within the Angiotensin-converting enzyme 2 (ACE2)-binding surface and consequently compromise the immune protection from vaccination (2, 18–20). Therefore, new generations of vaccines based on conservative sequences within regions that are responsible for the RBD–ACE2 interaction surface are urgent and necessary.

In addition to antibody-mediated neutralization, recent studies have shown that SARS-CoV-2 may be susceptible to cellular immunity. Rapid adaptive T-cell responses in some patients may account for their markedly lightened disease severity (21). Simultaneously activated humoral immunity and cellular immunity have been shown to have effective protection against SARS-CoV-2 attack in animals and, in some cases, can significantly increase the antigen-specific immune memory and duration (22). It would be of particular importance to address whether the inclusion of SARS-CoV-2-specific T-cell epitope could benefit the classic design of COVID-19 vaccines in terms of enhancing durable efficiency for the neutralization and clearance of currently circulating SARS-CoV-2 mutant strains (23–25).

Here, we characterize a potential SARS-CoV-2 vaccine candidate RBD9.1, which is a specific linear peptide shown in our previous report (23–25). The binding capability of COVID-19 convalescent serum to RBD9.1 is shown to be positively correlated to its neutralizing capacity. RBD9.1 is found to be located within a relatively conserved region of RBD. Immunization study demonstrated that in contrast to the compromised protection of the RBD-immunized group, the mouse sera from the RBD9.1 group exhibited sustained neutralizing efficacy against the circulating SARS-CoV-2 variant strains as the wild-type strain. Furthermore, RBD9.1 can activate the P45-specific CD8^+^ T-cell response in mice and generate a long-term protection with both humoral and cellular immunity. The peptide candidate presented in our study may shed light on optimizing the COVID-19 vaccine design to better fulfill the growing need of global protection against the newly emerged SARS-CoV-2 variants.

## Results

### Convalescent Sera With High Binding Ability to RBD9.1 Exhibited Marked Neutralizing Capability Against SARS-CoV-2 Pseudovirus

**Figure 1:**
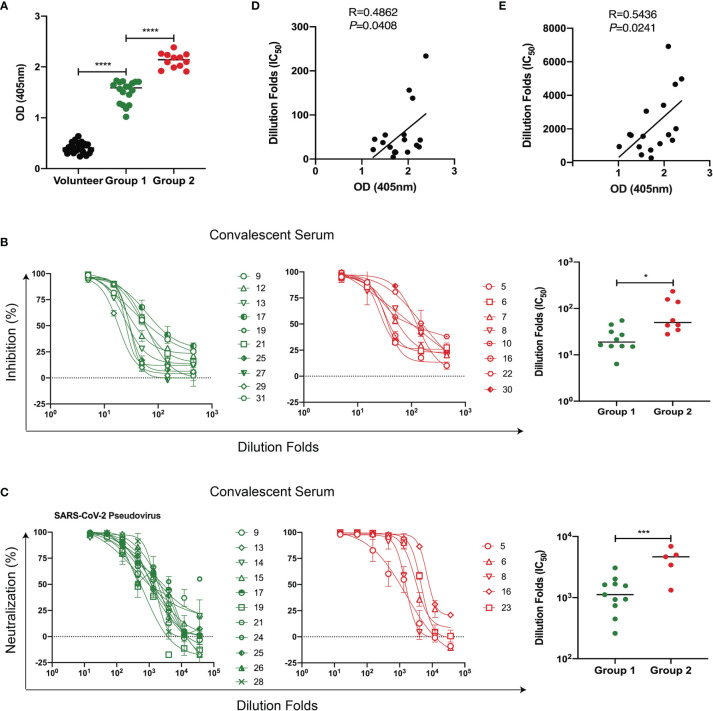
Convalescent sera with high binding ability to RBD9.1 exhibited marked neutralizing capability against severe acute respiratory syndrome coronavirus 2 (SARS-CoV-2) pseudovirus. **(A)** Human convalescent serum collected from recovered SARS-CoV-2 subjects (N = 31) and serum from healthy volunteers (N = 20) were analyzed for RBD9.1 binding capability. Different groups represented convalescent serum samples with a low (Group 1) or a high (Group 2) amount of RBD9.1-specific antibodies. **(B)** Receptor-binding domain (RBD)–hACE2 interaction inhibition titers (N = 18) and **(C)** SARS-CoV-2 pseudovirus neutralization titers (N = 16) were analyzed based on patients from Group 1 and Group 2. Each curve or points represented individual patients. Green: Group 1; red: Group 2. RBD9.1 binding antibodies were analyzed for the correlation with **(D)** RBD–hACE2 interaction inhibition titers (IC_50_) and **(E)** SARS-CoV-2 pseudovirus neutralization titers (IC_50_). Points or curve represent individual patients; horizontal lines indicate mean titers (MTs) of IC_50_ for each group ± SEM. *P < 0.05, ***P < 0.001, ****P < 0.0001.

From a pool of over 200 human monoclonal antibodies targeting the RBD protein from COVID-19 convalescent samples (6, 26), we have identified a linear region recognized by the most potent neutralizing antibody 58G6 (27). It covers a 20-amino acid (aa) sequence within the receptor-binding motif (RBM) and is named RBD9.1. First, we asked whether RBD9.1 was associated with the production of neutralizing antibodies in COVID-19 patients. ELISA results showed that RBD9.1-specific antibodies were detectable in all 31 convalescent sera but not in the sera from the volunteers (**Figure 1A**). Based on the binding capability of RBD9.1-specific antibodies within each sample, convalescent sera were divided into two groups, termed Group 1 (low) and Group 2 (high) (**Figure 1A** and **Supplementary Table S1**) in accordance with the average RBD9.1-specific antibody OD value of 31 samples. Detailed analysis of corresponding patient information showed that there was no correlation between the patient age or gender and the binding capability of the antibodies from individual samples (**Supplementary Figure S1A**). Moreover, the neutralizing capability of both groups was detected through RBD blocking assay and SARS-CoV-2 pseudovirus neutralization assay (**Supplementary Table S2**). Interestingly, we found that Group 2 demonstrated significantly higher ability in blocking SARS-CoV-2 pseudovirus as compared with Group 1 (**Figures 1B, C**). Correlation study confirmed that the amounts of RBD9.1 binding antibodies of individual samples were positively correlated with their neutralizing capability, represented by the RBD-hACE2 interaction inhibition titer (IC_50_) or the SARS-CoV-2 pseudovirus neutralization titer (IC_50_), respectively (**Figures 1D, E**). Meanwhile, no differences were observed for the neutralizing capability among convalescent sera from patients of different ages and genders (**Supplementary Figures 1B, C**). These results suggested that COVID-19 convalescent sera with high binding ability to RBD9.1 exhibited marked neutralizing capability against SARS-CoV-2 pseudovirus.

### Immunization With RBD9.1 Peptide Efficiently Induced Neutralizing Antibody Production in Mice

**Figure 2:**
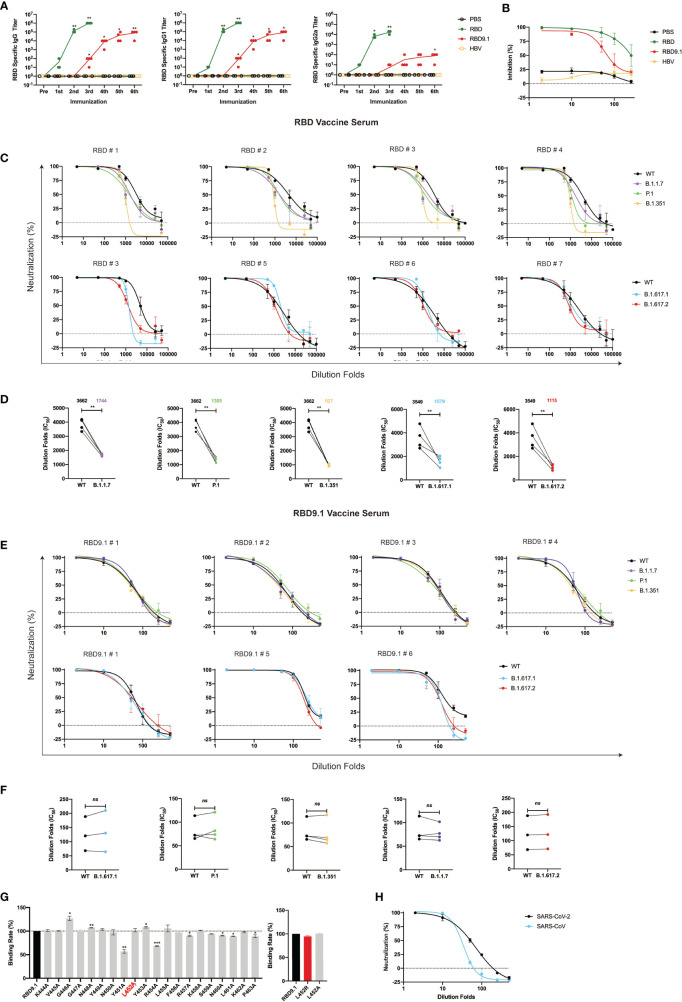
Immunization with RBD9.1 antigenic peptide efficiently induced neutralizing antibody production in Balb/c mice. **(A)** Receptor-binding domain (RBD)–binding immunoglobulin G (IgG; left), IgG1 (middle), and IgG2a (right) antibody titers tested by ELISA in mouse sera taken 10 days following each immunization. **(B)** RBD-blocking assay for severe acute respiratory syndrome coronavirus 2 (SARS-CoV-2) (wild-type) by mouse sera taken 10 days following the last dose of the RBD group or RBD9.1 group. Pseudovirus neutralization curves for SARS-CoV-2 (wild-type), B.1.1.7, P.1, B.1.351, B.1.617.1, and B.1.617.2 strains by mouse sera taken 10 days following the last dose of the RBD **(C, D)** and the RBD9.1 **(E, F)** vaccines. Comparison of neutralization titers between SARS-CoV-2 (wild-type) and five variant strains for the RBD and RBD9.1 vaccine serum: the Wilcoxon matched-pairs signed rank test was used for the analysis, and two-tailed *P* values were calculated; mean values were indicated above each column. Points represent individual mice in panels **(D, F)**. **(G)** Affinity for COVID-19 convalescent sera binding with RBD9.1 or 20 RBD9.1 point mutant peptides. Individually mutated amino acid was marked in red (**Supplementary Figure S1**). Serum binding affinity of each peptide was determined by the peptide exchange rates and shown by gray bars. S^L452R^ was shown by red bars. **(H)** RBD9.1 vaccines induced cross-neutralization to SARS-CoV. Serum samples were obtained 10 days following the sixth RBD9.1 antigenic peptide vaccine dose, with a dosing interval of 2–3 weeks. Data were presented as mean ± SEM. Representative data of two independent experiments were shown. *P < 0.05, **P < 0.01, ***P < 0.001; ns, non-significant.

Next, we determined whether RBD9.1 could induce immunogenic reactivity *in vivo*. The RBD9.1 peptide and another reported HBV peptide of 20 aa were coupled to RBD9.1 or hepatitis B virus (HBV) peptide to utilize for the immunization of Balb/c mice, with a dosing interval of 2–3 weeks (28) (**Supplementary Figures S2A, B**). With three rounds of peptide injection, we were able to detect the presence of RBD-specific immunoglobulin G (IgG) antibodies in the mouse serum (**Figure 2A**). The level of these antibodies appeared to be positively correlated with the course and duration of multiple rounds of peptide injections (**Figure 2A**). This was like the production of the hepatitis B surface antigen-specific antibodies in mice immunized with the HBV peptide (**Supplementary Figure S2D**). Additionally, we analyzed whether these antigen-specific IgG antibodies had any preference toward the Th1 or the Th2 subtype. ELISA results showed that the serum from mice immunized with RBD9.1 consisted of both RBD-specific IgG1 and IgG2a, while IgG1 exhibited approximately a 1,000-fold higher titer than IgG2a (**Figure 2A**). This suggested that a dominance of Th2-type antibodies was associated with the RBD9.1 immunization. Meanwhile, mice were immunized with highly purified RBD recombinant proteins that were expressed in FreeStyle 293F cells and isolated by affinity chromatography (**Supplementary Figure S2C**). As early as from a single shot, the full length of RBD was found to promote the production of RBD-specific IgG antibodies, preferentially the IgG1 subtype, which were at comparably higher titers than those from the RBD9.1 injections (**Figure 2A**). Furthermore, we determined whether these RBD-specific IgG antibodies from immunized mouse sera could block the interaction between RBD and hACE2. Serum samples obtained 10 days post the sixth RBD9.1 vaccination were applied in the RBD-blocking assay, we found that sera from both RBD- and RBD9.1-immunized mice exhibited competitive efficacy over hACE2 (**Figure 2B**). Although serum samples of the RBD group showed relatively increased levels of inhibitory effect at higher dilution rate compared to the serum from the RBD9.1 group, both could reach close to 100% competitive binding to RBD at high concentrations (**Figure 2B**). This indicated that the RBD9.1- and RBD-immunized mouse sera might contain neutralizing antibodies against SARS-CoV-2. Therefore, we moved on to characterize whether the RBD- and RBD9.1-immunized mouse sera had any neutralizing potential against the newly emerged SARS-CoV-2 variants. Through sequence comparison, we found that the sequence of RBD9.1 was mostly conserved among wild-type SARS-CoV-2 and five of the most contagious mutant strains, with exception of B.1.617.1 and B.1.617.2, which carried the S^L452R^ mutation within the RBD9.1 sequence (**Supplementary Figure S2G**). The pseudovirus neutralization experiment showed that the RBD sera had lower levels of geometric mean titers against all five variants than the wild-type pseudoviruses, with a range of 2.5- to 3.8-fold differences (**Figures 2C, D**). Importantly, the RBD9.1 antigenic peptide vaccine serum demonstrated sustained neutralizing capabilities for all five variants with comparative levels of IC50s as that of the wild-type SARS-CoV-2 pseudoviruses (**Figures 2E, F**). As negative controls, sera taken prior to RBD9.1 immunization or from the phosphate buffered saline (PBS)-injected group showed minimal or absent reactivity against any of the pseudoviruses (data not shown). A further evaluation of the key amino acids that might affect the binding affinity of the RBD-specific antibodies from the immunized mouse sera was performed. All the amino acids from RBD9.1 were individually substituted into alanine (A) (**Supplementary Figure S2E**). The ELISA results showed that the binding ability of the RBD9.1 serum to RBD9.1 was significantly decreased only with S^Y451A^ and S^Y454A^ (**Figure 2G**). The S^L452A^ and S^L452R^ mutation did not affect the binding of the RBD9.1 serum to this point mutated peptide, which was consistent with the sustained neutralization of these sera against B.1.617.1 and B.1.617.2 carrying the S^L452R^ mutation. Also, pseudovirus neutralization assay showed that the RBD9.1 serum could functionally block the binding of SARS-CoV to hACE2, though at a markedly lower level compared to its effect against SARS-CoV-2 (**Figure 2H** and **Supplementary Figure S2F**). It is worth mentioning that approximately 50% of the amino acid sequence corresponding to the RBD9.1 region was conserved between SARS-CoV-2 and SARS-CoV, including the S^Y451^ and S^Y454^ sites with functional importance for antigenic binding (**Figure 2H** and **Supplementary Figure S2F**). Together, the above results suggested that immunization with RBD9.1 could efficiently induce neutralizing antibody production in Balb/c mice, which remained functionally effective for the neutralization of newly emerged SARS-CoV-2 variant strains.

### RBD9.1 Efficiently Activated the Germinal Center Reaction

**Figure 3:**
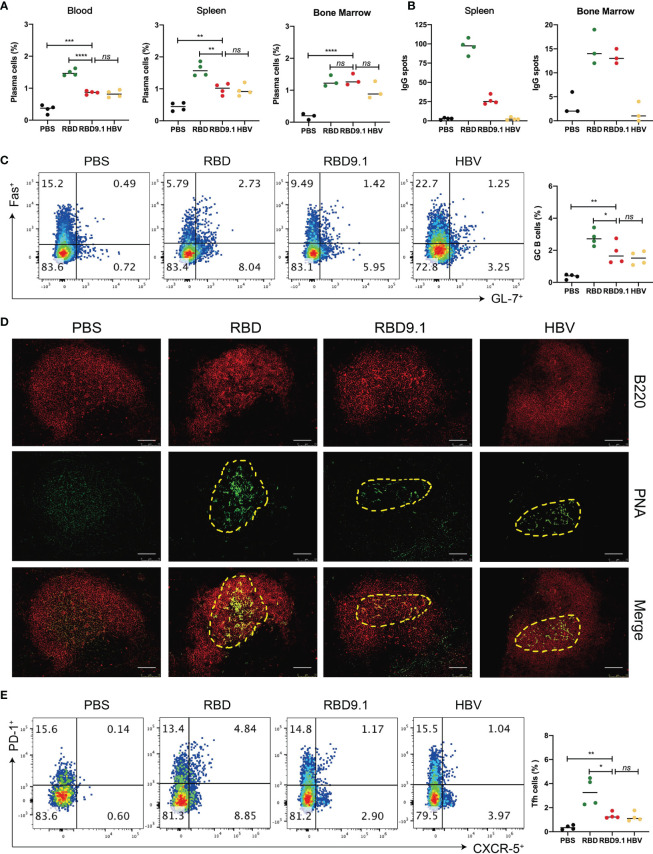
RBD9.1 efficiently activated the germinal center reaction. **(A)** The ratio of CD19^-^CD138^+^ plasma cells of lymphocytes in the blood, spleen, and bone marrow of mice on the seventh day after the last immunization. **(B)** Results were expressed as the numbers of receptor-binding domain (RBD)-specific immunoglobulin G (IgG) spots per 5 × 10^5^ splenocytes of each mouse subtracted the numbers from the corresponding DMSO groups. The stimulation with an equal volume of media was performed as the negative control. Data were representative of two independent experiments. **(C)** The percentages of Fas^+^ GL-7^+^ B cells from the splenocytes were shown in gated CD19^+^ B cells. **(D)** On the seventh day after the last immunization, the frozen tissue of the mouse spleen was stained with PNA (green) and B220 (red). The scale bar represented 75 μm, and the picture was analyzed by ImageJ software. **(E)** The percentages of PD-1^+^CXCR5^+^ T follicular helper (Tfh) cells from the splenocytes were shown in gated CD4^+^ T cells. Points represent individual mice. *P < 0.05, **P < 0.01, ***P < 0.001, ****P < 0.0001; ns, non-significant.

Successful production of high-affinity antibodies relies on the activation of the germinal center and the export of plasma cells post antigenic stimulation. Therefore, the accumulation of activated plasma cells after RBD9.1 immunization was studied. We examined the ratio of the CD19^-^CD138^+^ plasma cells in the total lymphocytes from the blood, spleen, and the bone marrow of immunized mice on the seventh day after the last injection. The results showed that RBD9.1 could promote a significant proportional increase of the CD19^-^CD138^+^ plasma cells in all three tissues relative to the PBS control at a similar level to the HBV group (**Figure 3A**). The RBD group showed a comparably higher level of CD19^-^CD138^+^ plasma cell accumulation than that of the RBD9.1 group in both the blood and the spleen, while no significant difference was found between these groups in the bone marrow (**Figure 3A** and **Supplementary Figure S3A**). Furthermore, ELISPOT experiment showed that RBD-specific plasma cells were only detectable in the RBD and the RBD9.1 groups, and the number of spots in the RBD group was markedly higher than that of the RBD9.1 group in the spleen but not in the bone marrow (**Figure 3B** and **Supplementary Figure S3B**).

Further analyses of the germinal center of each group showed that CD19^+^GL7^+^ Fas^+^ B cells could be detected in all immunized groups, except for PBS, while the RBD group was associated with a significantly higher number of these B cells than that of the RBD9.1 group (**Figure 3C**). Also, immunofluorescent staining of the germinal center B-cell marker Peanut hemagglutinin (PNA) showed that it was expressed at high levels in the splenic B cells of mice immunized with RBD9.1, RBD, or HBV peptide (**Figure 3D**). In parallel, we detected the proportion of CD4^+^CXCR5^+^PD-1^+^ T follicular helper (Tfh) in the spleen of immunized mice. Flow cytometry analysis revealed that these Tfh cells were expanded with RBD9.1 injection at a level like that of the HBV group but markedly lower than that of the RBD group (**Figure 3E**). Collectively, these results confirmed that RBD9.1 can effectively activate the humoral immunity by promoting the formation of germinal centers in mouse spleen and exporting RBD-specific plasma cells.

### RBD9.1 Induced CD4^+^ and P45-Specific CD8^+^ T-Cell Immune Responses

**Figure 4:**
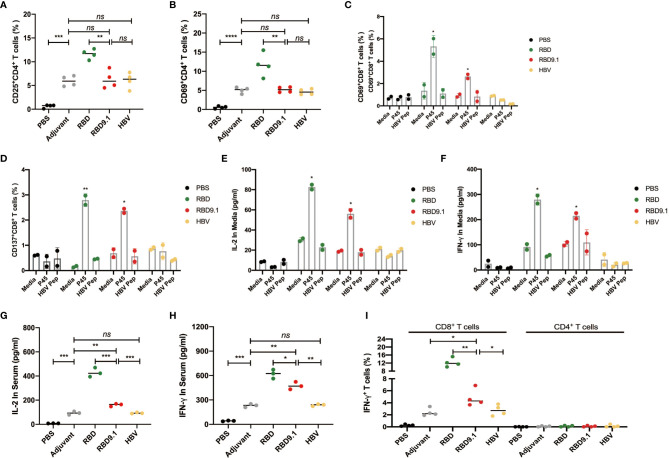
RBD9.1 induced CD4+ and S448-456-specific CD8+ T-cell immune responses. **(A)** The expressions of CD25 and CD69 **(B)** (gated on CD4^+^ T cells) were detected by flow cytometry on the seventh day after the last immunization. **(C)** The expressions of CD137 and CD69 **(D)** (gated on CD8^+^ T cells of immune mice) were detected by flow cytometry after 10 μg/ml P45 (S^448-456^) or HBV peptide stimulation for 24 h; normal media was used as negative control. **(E)** The quantities of interleukin (IL)-2 and interferon (IFN)-γ **(F)** in the supernatant were detected by ELISA after 10 μg/ml P45 (*S^448-456^*) or HBV peptide stimulation for 48 h; media or HBV peptide was used as the corresponding negative control. **(G)** IL-2 and IFN-γ **(H)** in the immunized serum were detected by ELISA on the 10th day after the last immunization. The expression of IFN-γ (gated on CD4^+^ or CD8^+^ T cells) was detected by flow cytometry on the 10th day after the last immunization. *P < 0.05, **P < 0.01, ***P < 0.001, ****P < 0.0001; ns, non-significant.

Since the antibody production from B cells requires the facilitation of CD4^+^ Tfh cells for antigenic recognition, we evaluated the CD4^+^ T-cell activation. Though higher than the PBS control, no apparent differences were observed for the expressions of CD25 and CD69 in the splenic CD4^+^ T cells among the RBD9.1, the HBV, or the adjuvant groups, suggesting that the induced CD4^+^ T-cell activation was an adjuvant effect (**Supplementary Figures S4A, B**). Consistent with the level of the germinal center activation, the highest percentages of CD25^+^ or CD69^+^ CD4^+^ T cells were found to be from the RBD-immunized group (**Figures 4A, B**).

To be noted, the sequence of RBD9.1 contains a 9-aa region (P45) that we have recently confirmed to be an HLA-A*24:02 CD8^+^ T-cell epitope (25). Therefore, we suspected that the RBD9.1 antigenic peptide might induce a P45-specific T-cell response. We verified whether P45 could be presented by the Balb/c mouse major histocompatibility complex (MHC)-I molecules, which were mainly H2-Kd and H2-Dd, with H2-Ld to a lesser extent. Through the prediction by IEDB (http://www.iedb.org) and NetMHCpan software (http://www.cbs.dtu.dk/services/NetMHCpan/), it was shown that P45 could potentially bind to the mouse HLA molecules, especially H2-Kd and H2-Dd (data not shown). With this, we stimulated the splenocytes isolated from the immunized mice by the P45 peptide for 24 h and examined the expression of T-cell activation marker CD69 and CD137. Flow cytometric analysis showed that a higher amount of activated CD8^+^ T cells were detected in both the RBD and RBD9.1 groups, whereas no differences were found between the two groups of these antigenic peptides (**Figures 4C, D** and **Supplementary Figures S5A, B**). In addition, the amounts of interleukin (IL)-2 and interferon (IFN)-γ secreted by the immunized splenocytes were both increased in the RBD9.1 and the RBD group after 48-h stimulation with P45 (**Figures 4E, F**). Moreover, the levels of IL-2 and IFN-γ in the serum of each immunized group were analyzed, and the results showed that both RBD and RBD9.1 could significantly enhance the production of these two cytokines relative to the HBV peptide or the PBS control (**Figures 4G, H**). Detailed study of the source of these cytokines by flow cytometry analysis revealed that IFN-γ was predominantly secreted by CD8^+^ T cells, which was consistent with the P45-specific T-cell reactivity identified above (**Figure 4I** and **Supplementary Figures S6A, B**). Overall, these data indicated that the RBD9.1 antigenic peptide could induce both CD4^+^ T cell and the P45-specific CD8^+^ T-cell immune response.

### RBD9.1 Induced Both Memory B Cells and T Cells

**Figure 5:**
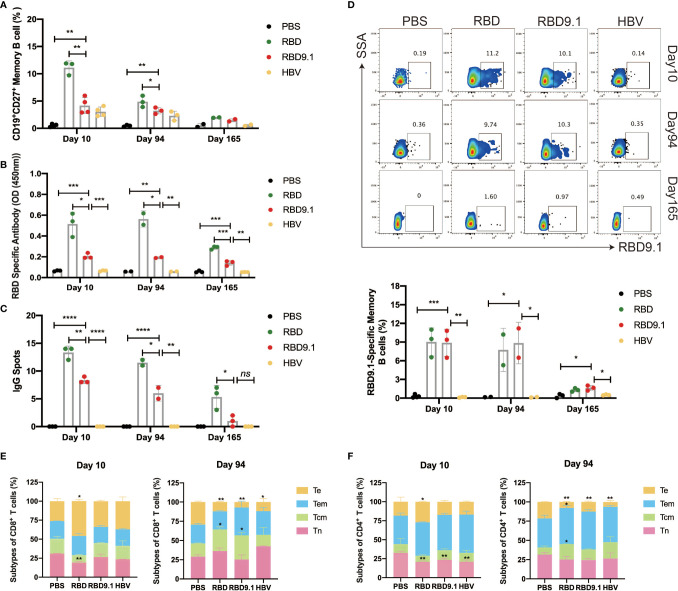
RBD9.1 induced both memory B cells and T cells. **(A)** The percentages of CD19^+^CD27^+^ memory B cells (gated on CD19^+^ B cells) from the splenocytes were detected by flow cytometry on the 10th, 94th, and 165th day after the last immunization. R848 (2 μg/ml) combined with 100 U mouse IL-2 was used to induce memory B cells to differentiate into plasma cells on days 10, 94, and 165 after the last immunization. **(B)** Receptor-binding domain (RBD)-specific immunoglobulin G (IgG) antibodies in the supernatant of 2 × 10^6^ splenocytes per ml were detected by ELISA. **(C)** Stimulation with R848 and IL-2 for 6 days, the ELISPOT picture showed the numbers of RBD-specific IgG spots per 5 × 10^5^ splenocytes of each mouse, subtracted those from the corresponding DMSO groups. The stimulation with an equal volume of media was performed as the negative control. Data were representative of two independent experiments. **(D)** RBD9.1-specific memory B cells (gated on CD19^+^CD27^+^ B cells) were detected by flow cytometry on days 10, 94, and 165 after the last immunization. **(E, F)** The ratio of Tn (CD62L^+^CD44^-^), Te (CD62L^-^CD44^-^), Tem (CD62L^-^CD44^+^), and Tcm (CD62L^+^CD44^+^) of CD8^+^ or CD4^+^ T cells on days 10 and 94 after the last immunization. *P < 0.05, **P < 0.01, ***P < 0.001, ****P < 0.0001; ns, non-significant.

To evaluate whether RBD9.1 immunization could form long-term immune memory, we assessed the levels of memory B and T cells on Days 10, 94, and 165. Flow cytometry analysis of the memory B-cell marker CD27 showed that the proportion of CD19^+^CD27^+^ B cells was significantly increased in all three immunized groups, compared to the PBS control, while the RBD group showed the highest level of elevation in memory B cells (**Figure 5A**). The enhanced CD19^+^CD27^+^ B-cell percentages were shown to decrease over time, but they could still be detected at a late time point of 94 days (**Figure 5A**). Furthermore, we assessed the long-term production of the RBD-specific memory B cells. The results from the antibody-secreting cell (ASC) ELISPOT assay and the ELISA assay showed that both RBD and RBD9.1 could efficiently induce the production of RBD-specific IgG antibodies, with the former at a relatively higher level, in the supernatant after costimulation with R848 and mouse IL-2 for 6 days (**Figure 5B** and **Supplementary Figure S7B**). There were no statistical differences between the levels of antibody productions of these two groups on the 10th and the 94th day, whereas sight reduction was observed in the number of RBD-specific IgG spots as well as the total antibody amounts after the last immunization on the 165th day (**Figure 5C** and **Supplementary Figure S7A**). Importantly, the presence of RBD9.1-specific memory B cells was associated with both the RBD and RBD9.1 groups at comparative levels, which were sustained over the period of the 94-day immunization course and decreased on Day 165 (**Figure 5D**).

Moreover, the proportions of T-cell subgroups in the CD4^+^ and CD8^+^ T cells on Days 10 and 94 after the last immunization were assessed (**Figures 5E, F** and **Supplementary Figures S7C, D**). Compared with those of the other three groups, the percentages of Tn in CD8^+^ T cells in RBD group significantly decreased on Day 10, along with a significant increase in Te subpopulation. Compared with that of the PBS group, the percentages of Tn in CD4^+^ T cells in all three groups significantly decreased on Day 10, while only the RBD group showed a significant increase in the Te subpopulation. No apparent changes were observed for the Tcm or Tem subgroups in CD4^+^ or CD8^+^ T cells in any immunized samples. On Day 94, the proportion of Tn and Tem in CD8^+^ did not alter in any sample. However, significantly higher Tcm proportions in CD8^+^ T cells were found in the RBD and RBD9.1 groups than that in the PBS group, which confirmed that RBD9.1 peptide promoted the information of CD8^+^ T-cell memory in mice after six rounds of immunization. This was accompanied by significantly reduced levels of Te of CD8^+^ T cells in the RBD, RBD9.1, and HBV groups compared with that of the PBS control. Meanwhile, it was observed that the percentages of Te in CD4^+^ T cells in the RBD, RBD9.1, and HBV groups all significantly decreased compared with the PBS control on Day 94. The level of Tcm was only found to be significantly increased in the RBD group, indicating that CD4^+^ T-cell memory might be specifically associated with RBD immunization. Together, these results demonstrated that RBD9.1 peptide could effectively establish long-term B-cell and T-cell immune memory.

### RBD9.1 Antigenic Peptide Induced Long-Term Protection

**Figure 6:**
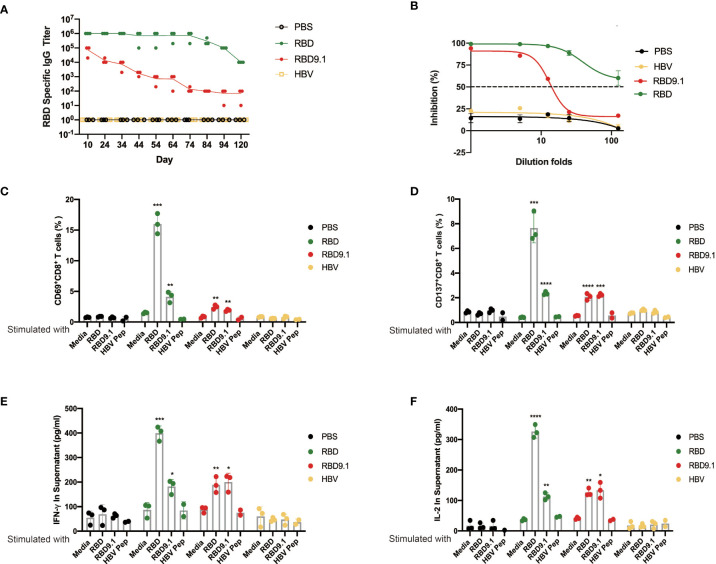
**(A)** RBD9.1 antigenic peptide induced long-term protection. Receptor-binding domain (RBD)–binding immunoglobulin G (IgG) antibody ELISA titers by mouse sera taken 10, 24, 34, 44, 54, 64, 74, 84, 94, and 120 days following the last immunization. **(B)** RBD-blocking assay for severe acute respiratory syndrome coronavirus 2 (SARS-CoV-2) (wild-type) by mouse sera taken 64 days following the last dose of RBD or RBD9.1. **(C)** The expressions of CD69 and CD137 **(D)** (gated on CD8^+^ T cells of immune mice) were detected by flow cytometry after 10 μg/ml RBD, RBD9.1, or HBV peptide stimulation for 24 h; normal medium was used as negative control. **(E)** The quantities of interleukin (IL)-2 and interferon (IFN)-γ **(F)** in the supernatant were detected by ELISA after 10 μg/ml of RBD, RBD9.1, or HBV peptide stimulation for 48 h; normal medium or HBV peptide was used as corresponding negative control. *P < 0.05, **P < 0.01, ***P < 0.001, ****P < 0.0001.

To verify the long-term protection with RBD9.1 immunization, an antibody titer follow-up study was performed. The results showed that the RBD-specific antibodies were still detectable 4 months post the last injection of RBD or RBD9.1, with the latter to a lower level (**Figure 6A**). Specifically, the RBD-blocking assay showed that the RBD-specific IgG antibodies from mouse sera 64 days post the last RBD9.1 immunization could block the interaction between RBD and hACE2 (**Figure 6B**).

Also, flow cytometry analysis was performed with the splenocytes stimulated with RBD9.1 *ex vivo*. We found that both RBD and RBD9.1 could induce the activation of CD8^+^ T cells, indicated by the elevated percentages of the CD69^+^CD8^+^ or CD137^+^CD8^+^ T cells (**Figures 6C, D** and **Supplementary Figures S8A, B**). Consistently, increased secretion of IFN-γ and IL-2 was detected with both RBD9.1 and RBD stimulation 94 days after the last immunization (**Figures 6E, F**). These results suggested that RBD9.1 immunization might provide long-term protection with sustained levels of RBD-specific antibodies and functionally durable CD8^+^ T-cell responses.

## Discussion

Currently, vaccination against COVID-19 has been promoted worldwide, although sustained protection against the newly emerged SARS-CoV-2 variant strains has been continuously challenged (29, 30). Therefore, a need for a vaccine candidate with cross-protection against SARS-CoV-2 strains with enhanced infectivity is raised, especially for those with mutations in the RBM region and resistant to serum neutralization. In this study, we found that RBD9.1 could be widely recognized by COVID-19 convalescent serum, with higher affinity for those with elevated neutralizing potency. Importantly, the RBD9.1-immunized mouse sera exhibited sustained neutralizing capability against five of the currently dominant variant strains as the wild-type SARS-CoV-2, including B.1.617.2 that carried a point mutation within the sequence of RBD9.1 (S^L452R^). Furthermore, we confirmed that the RBD9.1 antigenic peptide could induce a specific CD8^+^ T-cell response, as the sequence of RBD9.1 contained a previously identified T-cell epitope P45 (S^448-456^). Together, we provided a potential vaccine candidate, with a unique functional mechanism of activating both humoral and cellular immunity, as well as a long-term protective efficacy over SARS-CoV-2 variant strains.

The SARS-CoV-2 RBD has been a functional antigenic during the development of candidate vaccines for COVID-19 (31). However, undesired major immune responses have presented a great threat and largely limited its practical applications. The identification of the linear immunodominant RBD9.1 and the reactiveness of the sera from COVID-19 patients against it have confirmed that it might be an alternative expected to bypass certain limitations associated with the full length of RBD. Indeed, the serum from mice immunized with RBD9.1 contained a relatively lower proportion of RBD-specific non-neutralizing antibodies than that of the serum from RBD-immunized group. Reasonably, RBD9.1 is less effective than RBD in terms of the bulk of induced RBD-specific antibodies, which is largely due to the significant differences in the length and structural complexity between these antigenic peptides. Also, RBD9.1 could induce long-term protection of the body after immunization, even if the number of RBD-specific memory B cells is less than that of RBD. Independently, another study showed that a longer peptide with 46 aa (S^435-479^) covering the sequences of RBD9.1 (S^444-463^) could induce neutralizing protection in mice against authentic SARS-CoV-2 (32). To be noted, the neutralization titers of these two peptides were almost identical in the RBD-hACE2-blocking assay or the SARS-CoV-2 pseudovirus neutralization assay (data not shown). These suggested that RBD9.1 might be of advanced practical value for the design of *de novo* peptide-based vaccines for COVID-19.

As most of the currently available SARS-CoV-2 commercial vaccine products have been reported with significantly reduced neutralizing efficacy against mutant strains, public concern has risen for the post-vaccination protective outcome, particularly facing the global threat of B.1.617.2. In contrast to most reported vaccines, we found that the RBM mutations presented in five of the dominant SARS-CoV-2 variant strains did not seem to challenge the neutralizing capability of the RBD9.1-immunized sera. This is largely because the sequence of RBD9.1 is a relatively conserved region within the RBM. Further characterization for the binding ability of the neutralizing antibodies revealed two key residues within RBD9.1, for which there was no reported variant strain with corresponding point mutations so far. Coincidentally, with B.1.617 and B.1.617.2 containing a close point mutation (S^L452R^) in the RBD9.1 region, the immunized sera still exhibited effective neutralizing potency that was comparative to the wide type of the virus. Therefore, RBD9.1 may exhibit applicable importance with desirable protection from SARS-CoV-2, especially against mutant strains circulating at the current time. Majority of peptide-based vaccine products rely on the effective induction alone of humoral or cellular immune response post immunization (32–34). We have identified a rare case with RBD9.1, which is a linear region that could both induce the production of neutralizing antibodies and activate the CD8^+^ cytotoxic T cells in immunized mice. Particularly, we have proven that the inclusion of the previously reported immunodominant HLA-A*24:02-restricted peptide P45 (NYNYLYRLF) in the sequence of RBD9.1 was positively associated with enhanced production of functional cytokines essential for the antiviral effects of CD8^+^ cytotoxic T lymphocytes and the homologous assistance of CD4^+^ T lymphocytes (25, 35). It is of great importance because SARS-CoV-2 has been reported to be susceptible to cell-mediated immune responses, and COVID-19 patients who developed strong T-cell immunity are often correlated with mild symptoms. Therefore, RBD9.1-induced activation of cellular immunity in addition to humoral immunity might present a unique practical value during the development of COVID-19 vaccines. To be noted, although the point mutation S^L452R^ in B.1.617.2 was recently shown to largely impede the cytotoxic effect of the P45-specific CD8^+^ T cells, we confirmed that the neutralizing efficacy of RBD9.1-immunized sera was not affected against the corresponding pseudoviruses. This indicated that amino acid residues that were fundamental for the recognition of CD8^+^ T-cell receptor (TCR) might be different from those essential for the binding of neutralizing antibodies, and vaccine candidates like RBD9.1 that could functionally activate duo immune responses might provide clinical significance for SARS-CoV-2 prophylaxis.

One of the concerning side effects of COVID-19 vaccination was the antibody-dependent enhancement (ADE) effect (36, 37). For example, SARS-CoV-2 antibodies were found to especially induce ADE effects when inoculated with mutant strains containing the D617G mutation, which was shared by all the newly emerged mutant strains. According to the strategic enlightenment of SARS-CoV and Middle East respiratory syndrome coronavirus (MERS-CoV) vaccine development, short length of the vaccine peptide and high amounts of induced antibodies with neutralizing capacity were the two most important factors correlated with a reduced risk of ADE. Containing only 20 aa, RBD9.1 was correlated with high levels of neutralizing antibodies not only from COVID-19 convalescent patient samples but also in the immunized mouse sera. These might greatly reduce the likelihood of RBD9.1 in generating high levels of ADE, although detailed comparative studies are still needed. Meanwhile, the relatively short length of RBD9.1 has presented ideal opportunities for beneficial modifications, including the conjugation of polypeptides or the addition of SARS-CoV-2-specific CD4^+^ and/or CD8^+^ T-cell epitopes. Comprehensive preclinical evaluation of the immune response induced by RBD9.1 in advanced preclinical models, such as humanized ACE2 transgenic mice or rhesus monkeys, may provide valuable information for the evaluation and application of this vaccine candidate and is currently under study.

In conclusion, we presented a potential SARS-CoV-2 peptide vaccine candidate RBD9.1, with duo functional activations of humoral and cellular immunity, as well as generating long-term protective efficacy over SARS-CoV-2 variant strains. Our study provided practical information for the advancement of prophylaxis against COVID-19, especially with the currently circulating mainstream SARS-CoV-2 mutant strains.

## Materials and Methods

### Human SARS-CoV-2 Convalescent Serum Samples

We have complied with all relevant ethical regulations for work with human participants, and informed consent was obtained. This study has been approved by the ethics board of Chongqing Medical University, and the approval letter was included in the **Supplementary Information**. The 31 human convalescent serum samples from recovered SARS-CoV-2 patients were obtained from Chongqing, China. And these 31 convalescent patients have an average age of 45 years, and majority of them exhibited mild symptoms (**Supplementary Table S1**). All convalescent serum samples were heat inactivated at 56°C for 30 min before being used for analysis.

### Cell Lines

HEK 293T cells and 293F cells were purchased from the American Type Culture Collection (ATCC). HEK 293T cells and hACE2-293T cells were maintained in Dulbecco’s modified Eagle’s medium (Gibco™) supplemented with 10% fetal bovine serum (Gibco), 100 mg/ml streptomycin, and 100 units/ml penicillin at 37°C in 5% CO_2_. Splenocytes of mice were maintained in RPMI 1640 medium (Gibco™) supplemented with 10% fetal bovine serum (Gibco), 100 mg/ml streptomycin, and 100 units/ml penicillin at 37°C in 5% CO_2_.

### Plasmids

The codon-optimized gene encoding SARS-CoV-2-S (GenBank: QVE75681.1), SARS-CoV-2-S^B.1.1.7^ (GenBank: QHD43416), SARS-CoV-2-S^P.1^ (GenBank: EPI_ISL_2876136), SARS-CoV-2-S^B.1.351^ (GenBank: MZ314998), SARS-CoV-2-S^B.1.617.1^ (GISAID: EPI_ISL_2652002), and SARS-CoV-2-S^B.1.617.2^ (EPI_ISL_4299998) and with SARS-CoV-S C-terminal 19-aa deletion was synthesized and cloned into the *EcoR*I restriction sites of pMD2.G vector by Tsingke Biotechnology (Beijing, China). The PWPXL luciferase reporter vector (pWPXL-luciferase) constructed by N. Landau was provided by Prof. Cheguo Cai from Wuhan University (Wuhan, China). The VSV-G-expressing plasmid pMD2.G was provided by Prof. Ding Xue from Tsinghua University (Beijing, China). The expression plasmid for human ACE2 was obtained from GeneCopoeia (Guangzhou, China).

### Synthesis of Peptide and the Production and Purification of RBD Protein

The amino acid sequences of RBD9.1 and HBV peptide were shown in **Supplementary Figure S2**. The complex of peptides and Keyhole Limpet Haemocyanin (KLH) was synthesized by GenScript and used to immunize mice. The peptides were conjugated to KLH using a C-terminal cysteine residue by GenScript (order number: C695JFH190). To express the wild-type SARS-CoV-2 RBD protein (residues 334-526), residue 319-514 aa were cloned into a mammalian expression vector PcDNA 3.4 upstream of a mouse IgGκ signal peptide and downstream of an AviTag and a 6× His tag. SARS-CoV-2 RBD recombinant protein was expressed in FreeStyle 293F cells (Thermo Fisher Scientific) for 7 days and isolated by affinity chromatography using a HisTrap column (GE Healthcare).

### Mice and Immunization Strategy

Six-week-old female Balb/c mice were supplied by the Animal Center of Chongqing Medical University (Chongqing, China) and raised under specific pathogen-free (SPF) standard conditions. Animals were housed in groups of up to 4–5 mice/cage at 18°C–24°C ambient temperature and 40%–60% humidity. Mice were fed a 20% protein diet and maintained on a 12-h light/dark cycle. Food and water were available *ad libitum*. Mice were divided into PBS, Adjuvant, HBV, RBD9.1, and RBD groups (N = 8–10, see **Supplementary Table S3**). Here, 50 μg RBD or peptide was dissolved in 100 µl PBS and then formulated in Freund’s complete adjuvant or Freund’s incomplete adjuvant (Sigma) at a ratio of 1.2:1. See **Supplementary Figure S2B** for the immunization strategy. Three subcutaneous immunizations were administered in RBD group (at Weeks 0, 2, and 4). Six subcutaneous immunizations were administered in PBS, Adjuvant, HBV, and RBD9.1 group (at Weeks 0, 2, 4, 6, 9, and 12). On day 10 after each immunization, tail vein blood was collected and immediately used for antibody analysis.

### Peptide ELISA

Peptide ELISA was performed with synthesized peptides (Genescripts). These peptides were tethered by N-terminal biotinylated linker peptides (biotin-ahx). The RBD9.1 amino acid residues were selected and mutated to alanine and synthesized by Genescripts (Wuhan, China). Here, 50 μl synthesized peptides were added to the streptavidin-coated 384-well plate in duplets to make a final concentration of 5 μg/ml. The plates were incubated for 2 h at room temperature (RT). After washing, the plates were blocked with Protein-Free Blocking Buffer (Pierce) at RT for 1 h and incubated with 50 µl testing serum with 1,000-fold dilution at RT for another 1 h. Reacted human SARS-CoV-2 convalescent serum was detected using ALkaline Phosphatase (ALP)-conjugated Goat F(ab’)2 Anti-Human [IgG (Fab’)2] secondary antibody (Abcam, ab98532, 1:2,000). Reacted mouse serum was detected using horseradish peroxidase (HRP)-conjugated Goat Anti-Mouse IgG H&L secondary antibody (Abcam, ab6789, 1:10,000). Peptide exchange rates (%) = [(A-Blank)]/(P-Blank) × 100, where A is the OD signal of 1,000-fold diluted serum binding to RBD9.1 peptide with single point mutant, and P is the OD signal of 1,000-fold diluted serum binding to RBD9.1 peptide (Positive control).

### Serum ELISA

RBD-specific IgG, IgG1, and IgG2a antibodies in mouse serum were detected by ELISA. Here, 20 μl RBD protein (Sinobiological) were added to the 384-well plate in duplets to make a final concentration of 3 μg/ml. The plates were incubated overnight at 4°C. After washing, the plates were blocked with blocking buffer [5% bovine serum albumin (BSA) plus 0.05% Tween 20] at 37°C for 1 h and incubated with 20 µl testing mouse serum with 10-fold serial dilutions at 37°C for 30 min. Reacted mouse serum was detected using HRP-conjugated Goat Anti-Mouse IgG H&L secondary antibody (Abcam, ab6789, 1:10,000), HRP-conjugated Goat Anti-Mouse IgG1 H&L (Bethyl, A90-105P, 1:10,000), and HRP-conjugated Goat Anti-Mouse IgG2a H&L (Bethyl, A90-107P, 1:10,000).

### IgG ELISPOT

Mouse splenocytes were stimulated with 2 μg/ml R848 (Sigma) and 100 U/ml mouse IL-2 (PeproTech) for 6 days to induce memory B cells to differentiate into plasma cells. IgG ELISPOT assays were performed as reported and with minor modification. Here, 35% alcohol with sterile water was used to activate the ELISPOT plates (Millipore) for less than 1 min, and the liquid was discarded. Then, 50 μl RBD (Sinobiological) were added to the plates in duplets to make a final concentration of 10 μg/ml overnight at 4°C. Then, 5 × 10^5^ splenocytes were seeded per well in ELISPOT plates and stimulated for 36 h with RBD9.1 peptide (10 μM each). Stimulation with an equimolar volume of media was performed as the negative control, and RBD was used as the positive control. Subsequently, the plates were developed with HRP-conjugated Goat Anti-Mouse IgG H&L secondary antibody (MabTech, 1:1,000). IgG spots were quantified with the AID ELISPOT Reader (AID, Germany). To quantify positive peptide-specific responses, results were expressed as the numbers of RBD-specific IgG spots per 5 × 10^5^ splenocytes of each mouse. IgG spots = (peptide-stimulated well # 1 - unstimulated well # 1) + (peptide-stimulated well # 2-unstimulated well # 2)/2.

### Competitive ELISA

Here, 20 μl RBD-mfc protein (Sinobiological) were added to the 384-well plate (Corning) in duplets to make a final concentration of 0.2 μg/ml overnight at 4°C. Then, the plates were blocked for 1 h with blocking buffer (5% BSA plus 0.05% Tween 20). Next, 20 μl per well mouse serum with 5-fold serial dilutions were added to plates incubated at 37°C for 40 min followed by same volume with 0.2 μg/ml ACE2-his protein (Sinobiological) at 37°C for another 40 min. After washing, Goat Anti-Mouse IgG H&L secondary antibody (Abcam, ab6789, 1:10,000) was incubated with the plate at RT for 30 min. Plates were added with TMB (MabTech) and stopped with 1 mol/L HCl and followed with quantification detection. The half-maximal inhibitory concentration (IC_50_) was determined by using four-parameter logistic regression. The percentage of inhibition was calculated as follows: % inhibition = [(A-Blank)-(P-Blank)]/(A-Blank) × 100, where A is the maximum OD signal of RBD binding to ACE2-his when no serum was present, and P is the OD signal of RBD binding to ACE2-his in the presence of serum at a given dilution. The IC_50_ of a given serum sample was defined as the reciprocal of the dilution where the sample shows 50% competition. For human samples, the selection criteria were available for additional testing.

### Immunofluorescence

The spleen of immunized mice was separated on day 7 after the final immunization and embedded in optimal cutting temperature (OCT) compound (SAKURA). The tissues were frozen with liquid nitrogen before sectioning (7 µm) on a cryostat. After being fixed in cold acetone and blocked with 5% FBS in PBS at RT for 1 h, the sections were incubated with Biotinylated PNA (VECTOR, 1:100) overnight at 4°C. DyLight 488 Streptavidin (BioLegend, 1:100) was used as the secondary antibody at RT for 1 h followed with Alexa Fluor647-conjugated anti-mouse CD45R (BioLegend, 1:150) at RT for 1 h. After staining, the sections were scanned under a Pannoramic SCAN instrument (3DHISTECH, Hungary).

### Cytokine Assay

The splenocytes were separated from immunized mice on day 7 or day 94 after the final immunization and resuspended in complete RPMI-1640 supplemented with 10% FBS, 100 mg/ml streptomycin, and 100 units/ml of penicillin. For *in vitro* stimulation, a total of 1 × 10^6^ splenocytes were incubated with RBD9.1 or RBD (10 μmol) in 200 μl of complete RPMI-1640 for 48 h at 37°C in a humidified atmosphere containing 5% CO_2_. Splenocytes stimulated with Phorbol 12-myristate13-acetate/ionomycin (PMA/I) (Sigma-Aldrich) served as positive controls. The cytokines IFN-γ and IL-2 in media or serum were detected by IFN-γ ELISA kit (BioLegend) and IL-2 ELISA kit (BioLegend), respectively, according to the manufacturer’s instructions.

### Flow Cytometric Analysis

Lymphocytes from blood, spleen, and bone marrow of immunized mice were harvested on day 7 after the final immunization and analyzed by flow cytometry. Dead cells were excluded by viability dye staining, and adherent cells were excluded by SSC/A and SSC/H gating analysis. Cells were analyzed by a BD LSRFortessa™ Flow Cytometry (BD Biosciences, USA). Data were acquired and analyzed by FlowJo version 10.5.2. LIVE/DEAD™ Fixable Dead Cell Stain Kits (Invitrogen) were used for viability dye staining. For surface staining, splenocytes were stained with the following antibodies: APC anti-mouse CD19 (Clone: 1D3/CD19, BioLegend), PE anti-mouse CD138 (Syndecan-1) (Clone: 281-2, BioLegend), FITC anti-mouse/rat/human CD27 (Clone: LG.3A10, BioLegend), PE anti-mouse CD279 (PD-1) (Clone: 29F.1A1, BioLegend), PE/Cyanine7 anti-mouse CD4 (Clone: GK1.5, BioLegend), and APC/Cyanine7 anti-mouse CD185 (CXCR5) (Clone: L138D7, BioLegend) for Tfh cell analysis; with FITC anti-mouse/human GL7 Antigen (Clone: GL7, BioLegend), PerCP/Cyanine5.5 anti-mouse CD95 (Fas), (Clone: SA367H8, BioLegend), and anti-B220-PerCP-Cy5.5 (Clone: RA3-6B2, BD Pharmingen™) mAb for GC B-cell analysis. Alexa Fluor 700 anti-mouse CD3 (Clone: 17A2, BioLegend), PE anti-CD8a-200 (Clone: 53-6.7, BioLegend), Brilliant Violet 605™ anti-mouse CD69 (Clone: H1.2F3, BioLegend), FITC anti-mouse CD107a (LAMP-1) (Clone: 1D4B, BioLegend).

### Production and Titration Detection of Pseudovirus

pVSVG expressing SARS-CoV-2 S protein was constructed using the packaging plasmid (VSV-G pseudotyped ΔG-luciferase). It encoded either the S protein of SARS-CoV-2, B.1.1.7, P.1, B.1.351, B.1.617.1 and B.1.617.2 was generated. Lenti-X293T cells were grown to 70% confluency before transfection with VSV-G pseudotyped ΔG-luciferase, pWPXL and pSPAX2. These cells were cultured overnight at 37°C with 5% CO_2_. Dulbecco’s modified Eagle’s medium (DMEM) supplemented with 5% fetal bovine serum and 100 IU/ml of penicillin and 100 μg/ml of streptomycin was added to the inoculated cells, which were cultured overnight for 48 h. The supernatant was harvested, filtered by 0.45-μm filter, and centrifuged at 300 g for 7 min to collect the supernatant, then aliquoted and stored at -80°C. The titers of the pseudoviruses were detected by Lenti-X qRT-PCR Titration Kit (Takara) according to the manufacturer’s instructions.

### Pseudovirus Neutralization Assay

Pseudovirus and mouse serum were generated as described above. The 50-μl serial diluted mouse serum was incubated with pseudovirus (1 × 10^9^ copies/ml) at 37°C for 1 h. These pseudovirus–serum mixtures were added to ACE2 expressing Lenti-X293T cells (hACE2-293T). After 72 h, the luciferase activities of infected hACE2-293T cells were measured by the Bright-Luciferase Reporter Assay System (Promega). Relative luminescence unit (RLU) of Luc activity was detected using the ThermoFisher LUX reader. All experiments were performed at least three times and expressed as means ± SEM. Half-maximal inhibitory concentrations (IC_50_) of dilution folds were calculated using the four-parameter logistic regression in GraphPad Prism 8.0. For human samples, the selection criteria were available for additional testing.

### Statistical Analysis

Statistical analyses of the data were performed using GraphPad Prism version 8.0 software. Quantitative data in histograms and line charts were presented as mean ± SEM. Statistical significance was determined using ANOVA for multiple comparisons. Then, the pairwise comparison between multiple groups was performed using ANOVA *post-hoc* analysis; Bonferroni test method was selected at 0.05 level. Student’s t-tests were applied to compare two groups, and Spearman’s analyses were used for correlations, as shown in the figures. All statistical tests were two-tailed. A *P* value of less than 0.05 was considered significant.

## Data Availability Statement

The original contributions presented in the study are included in the article/**Supplementary Material**. Further inquiries can be directed to the corresponding author.

## Ethics Statement

The studies involving human participants were reviewed and approved by the Ethics Board of Chongqing Medical University. The patients/participants provided their written informed consent to participate in this study. The animal study was reviewed and approved by the Laboratory Animal Ethics Committee of Chongqing Medical University.

## Author Contributions

AJ, FG, and JH contributed to the conception and design of the studies and reviewed data over the course of the studies. FG, TL, CH, MS, FL, and SM performed experiments and analyzed data or generated reagents supporting the studies. FG and JH managed the animal procurement and shipping. FG contributed to the first drafts of the article. TL, AJ, and WW edited sections of the article. All authors contributed to the article and approved the submitted version.

## Funding

The study was supported by the Emergency Project for Novel Coronavirus Pneumonia from Chongqing Medical University.

## Conflict of Interest

The authors declare that the research was conducted in the absence of any commercial or financial relationships that could be construed as a potential conflict of interest.

## Publisher’s Note

All claims expressed in this article are solely those of the authors and do not necessarily represent those of their affiliated organizations, or those of the publisher, the editors and the reviewers. Any product that may be evaluated in this article, or claim that may be made by its manufacturer, is not guaranteed or endorsed by the publisher.

## Supplementary Material

Supplementary Figure 1 Detection of Neutralizing Antibodies in Human SARS-CoV-2 Convalescent Sera. **(A)** RBD9.1 binding antibody titers (N=31), RBD-hACE2 interaction inhibition titers **(B)** (N=18) and SARS-CoV-2 pseudovirus neutralization titers **(C)** (N=16) were analyzed based on gender and age in recovered SARS-CoV-2 subjects respectively. Points represent individual patients; ns represents non-significant.

Supplementary Figure 2 Immunization with RBD9.1 antigenic peptide efficiently induced neutralizing antibody production in Balb/c mice. **(A)** RBD9.1 and HBV peptide amino acid sequence information. **(B)** RBD9.1, RBD and HBV peptide immunization strategy. **(C)** SARS-CoV-2 RBD recombinant protein expressed in FreeStyle 293F cells and separated by affinity chromatography were verified by silver stain. **(D)** The production of HBV-specific antibodies from mice immunized with HBV peptide. **(E)** RBD9.1 sequence, with single point mutations (red) to alanine. **(F)** Sequence alignment for the RBD proteins of SARS-CoV-2 and SARS-CoV. The RBM region was marked in red, and the same amino acid sequence of the RBD protein of SARS-CoV-2 and SARS-CoV was marked in light blue. The amino acid sequence of RBD9.1 in SARS-CoV-2 RBD is marked with green dots. **(G)** Sequence alignment within the amino acid sequence of RBD9.1 of WT, P.1, B.1.1.7, B.1.351, B.1.617.1 and B.1.617.2 strains.

Supplementary Figure 3 Related to **Figure 3**. **(A)** Flow cytometric diagram of the proportion of CD19^-^CD138^+^ plasma cells. **(B)** ELISPOT results of RBD-specific plasma cells in the spleen and bone marrow of mice after last immunization. Results were expressed as the numbers of RBD-specific IgG spots per 5 × 10^5^ splenocytes of each mouse, subtracted those from the corresponding DMSO groups. The stimulation with an equal volume of media was performed as the negative control. Data were representative of two independent experiments.

Supplementary Figure 4 Related to **Figure 4**. The expression of CD25 **(A)** and CD69 **(B)** (gated on CD4+ T cells) on the 7th day after the last immunization.

Supplementary Figure 5 Related to **Figure 4**. The expression of CD137 **(A)** and CD69 **(B)** (gated on CD8^+^ T cells) after 10 μg/mL P45 or HBV peptide stimulation for 24 hours, normal media was used as negative control.

Supplementary Figure 6 Related to **Figure 4**. The expression of IFN-γ gated on CD8^+^ T cells **(A)** and on CD4^+^ T cells **(B)** on the 10th day after the last immunization.

Supplementary Figure 7 Related to **Figure 5**. **(A)** Stimulation with R848 and IL-2 for 6 days, the ELISPOT picture showed the number of RBD-specific IgG spots per 5 × 10^5^ splenocytes of each mouse. **(B)** The binding abilities of 10-fold serially diluted mouse serum to SARS-CoV-2 RBD recombinant protein. The ratio of Tn (CD62L^+^ CD44^-^), Te (CD62L^-^ CD44^-^), Tem (CD62L^-^ CD44^+^) and Tcm (CD62L^+^ CD44^+^) of CD8^+^ **(C)** or CD4^+^ T **(D)** cells on day 10 and day 94 after the last immunization.

Supplementary Figure 8 Related to **Figure 6**. The expression of CD69 **(A)** and CD137 **(B)** (gated on CD8^+^ T cells of immune mice) were detected by flow cytometry after 10 μg/mL RBD, RBD9.1 or HBV peptide stimulation for 24 hours, normal media was the negative control.

Supplementary Table 1 The information of Groups and OD 405nm were listed in **Supplementary Table 1**. And the threshold for grouping is 1.788, in accordance with the average OD value of 31 samples.

Supplementary Table 2 The clinical information and RBD-hACE2 interaction inhibition titers (IC_50_) and SARS-CoV-2 pseudovirus neutralization titers (IC_50_) were listed in **Table 2**.

Supplementary Table 3 The information of immunized mice was listed in **Table 3**.

Supplementary Table 4 The sequence information of SARS-COV-2 strains during RBD9.1 area were listed in **Table 4**.
