# Supplementary figures and images for: A Highly Conserved Peptide Vaccine Candidate Activates Both Humoral and Cellular Immunity Against SARS-CoV-2 Variant Strains
Source: Front Immunol. 2021 Dec 7;12:789905. doi: 10.3389/fimmu.2021.789905 (PMC8688401; doi:10.3389/fimmu.2021.789905)

# Supplemental Figure.1

A.

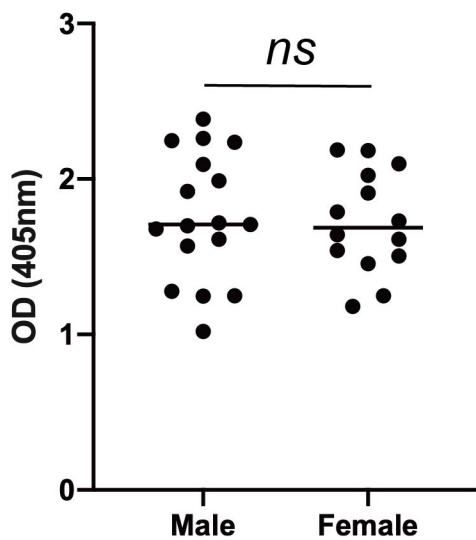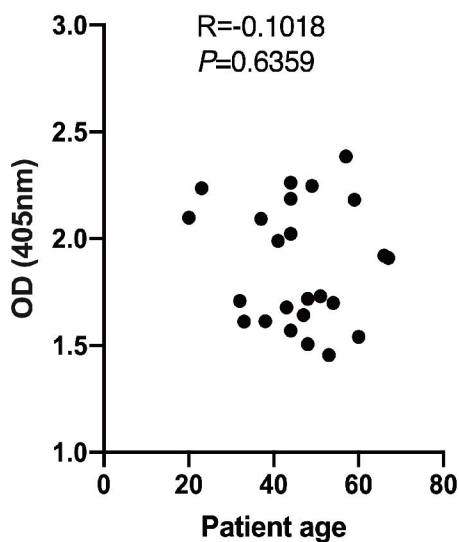

B.

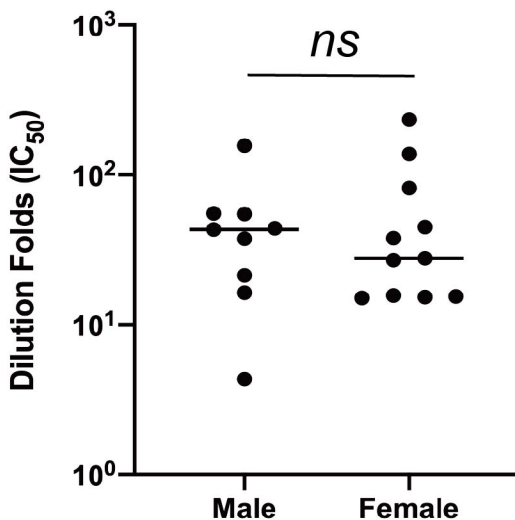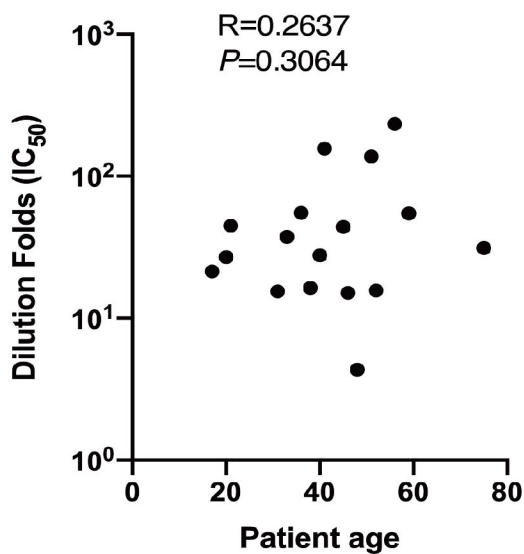

C.

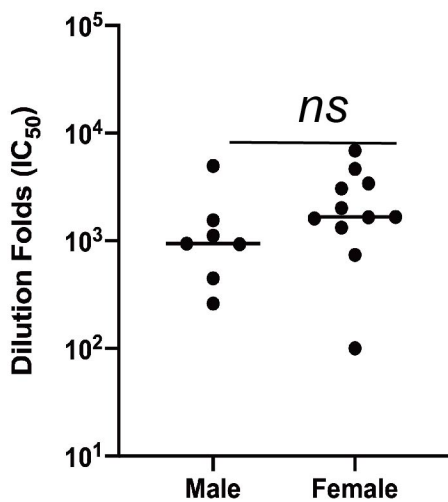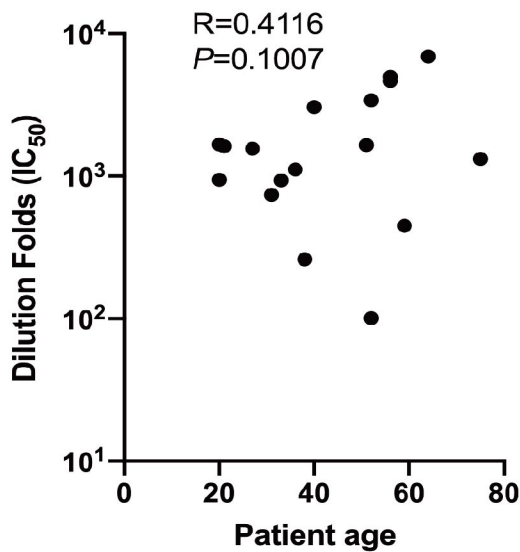

Supplement: Supplementary Figure 1 — Detection of Neutralizing Antibodies in Human SARS-CoV-2 Convalescent Sera. (A) RBD9.1 binding antibody titers (N=31), RBD-hACE2 interaction inhibition titers (B) (N=18) and SARS-CoV-2 pseudovirus neutralization titers (C) (N=16) were analyzed based on gender and age in recovered SARS-CoV-2 subjects respectively. Points represent individual patients; ns represents non-significant. [file Image_1.pdf]

# Supplemental Figure 3.

A.

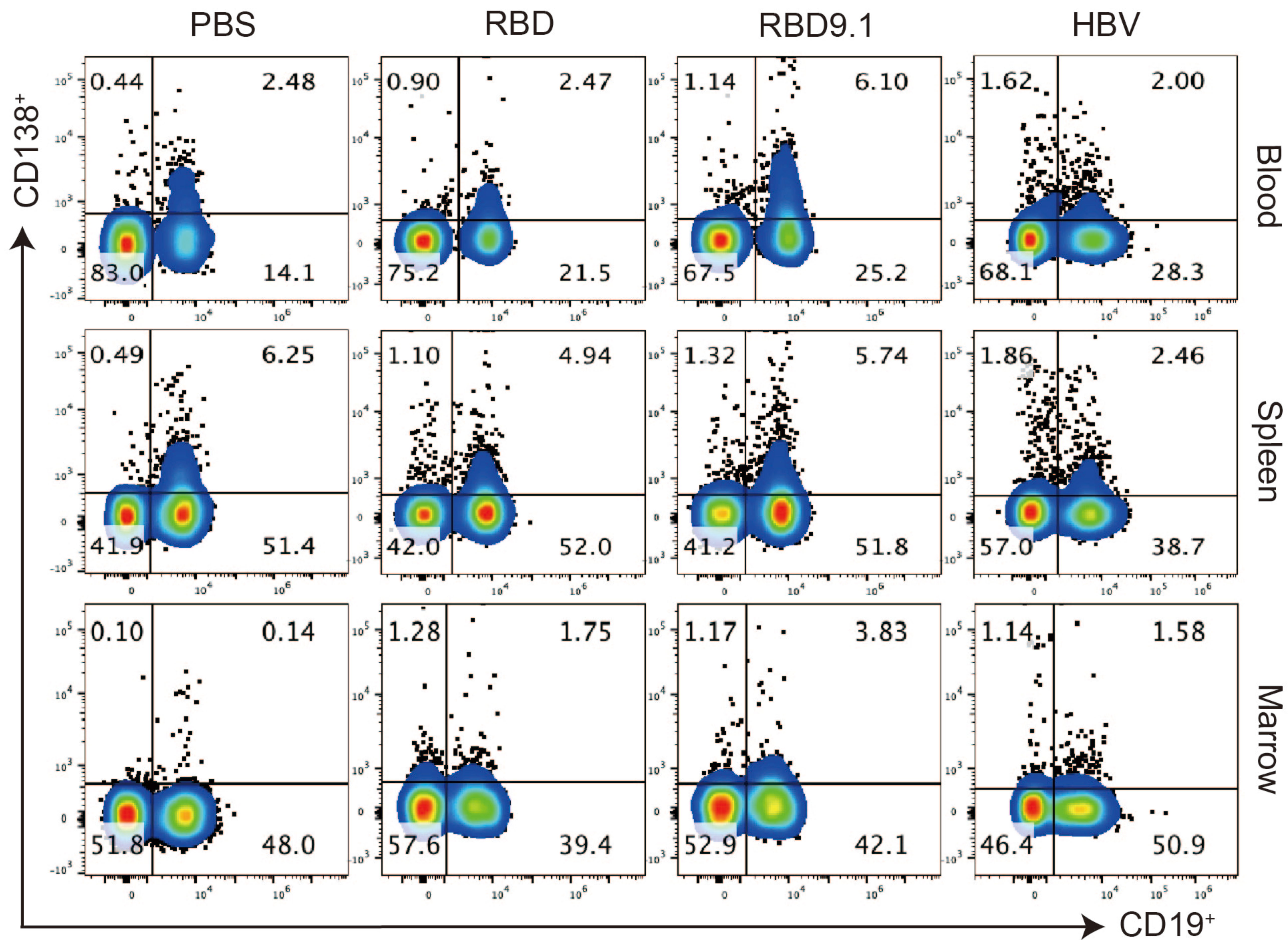

B.

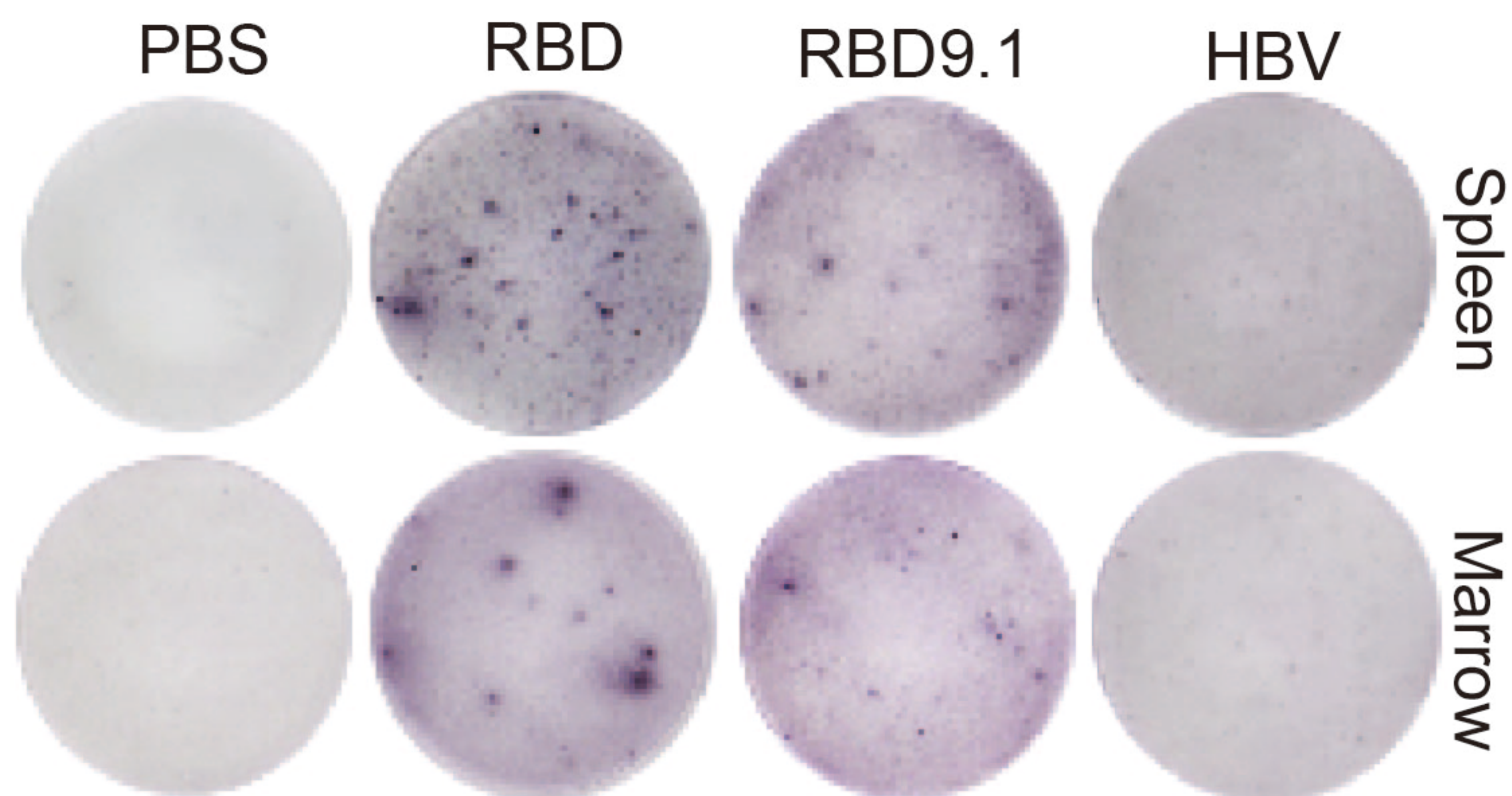

Supplement: Supplementary Figure 3 — Related to Figure 3 . (A) Flow cytometric diagram of the proportion of CD19-CD138+ plasma cells. (B) ELISPOT results of RBD-specific plasma cells in the spleen and bone marrow of mice after last immunization. Results were expressed as the numbers of RBD-specific IgG spots per 5 × 10^5^ splenocytes of each mouse, subtracted those from the corresponding DMSO groups. The stimulation with an equal volume of media was performed as the negative control. Data were representative of two independent experiments. [file Image_3.pdf]

Supplementary Figure.4

A.

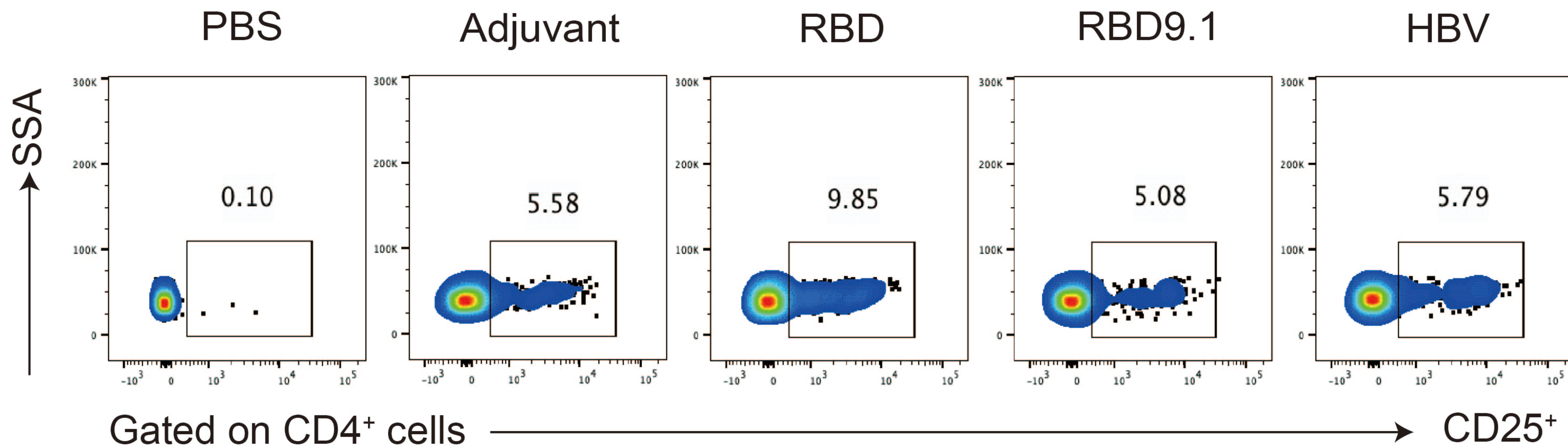

B.

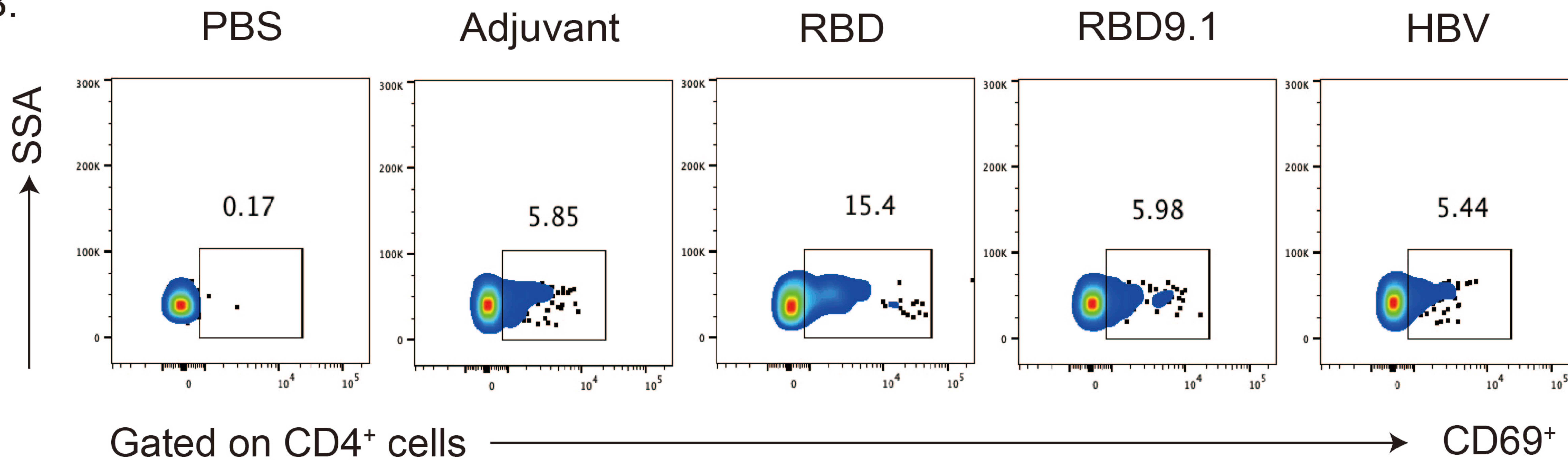

Supplement: Supplementary Figure 4 — Related to Figure 4 . The expression of CD25 (A) and CD69 (B) (gated on CD4+ T cells) on the 7th day after the last immunization. [file Image_4.pdf]

Supplementary Figure.5.

A.

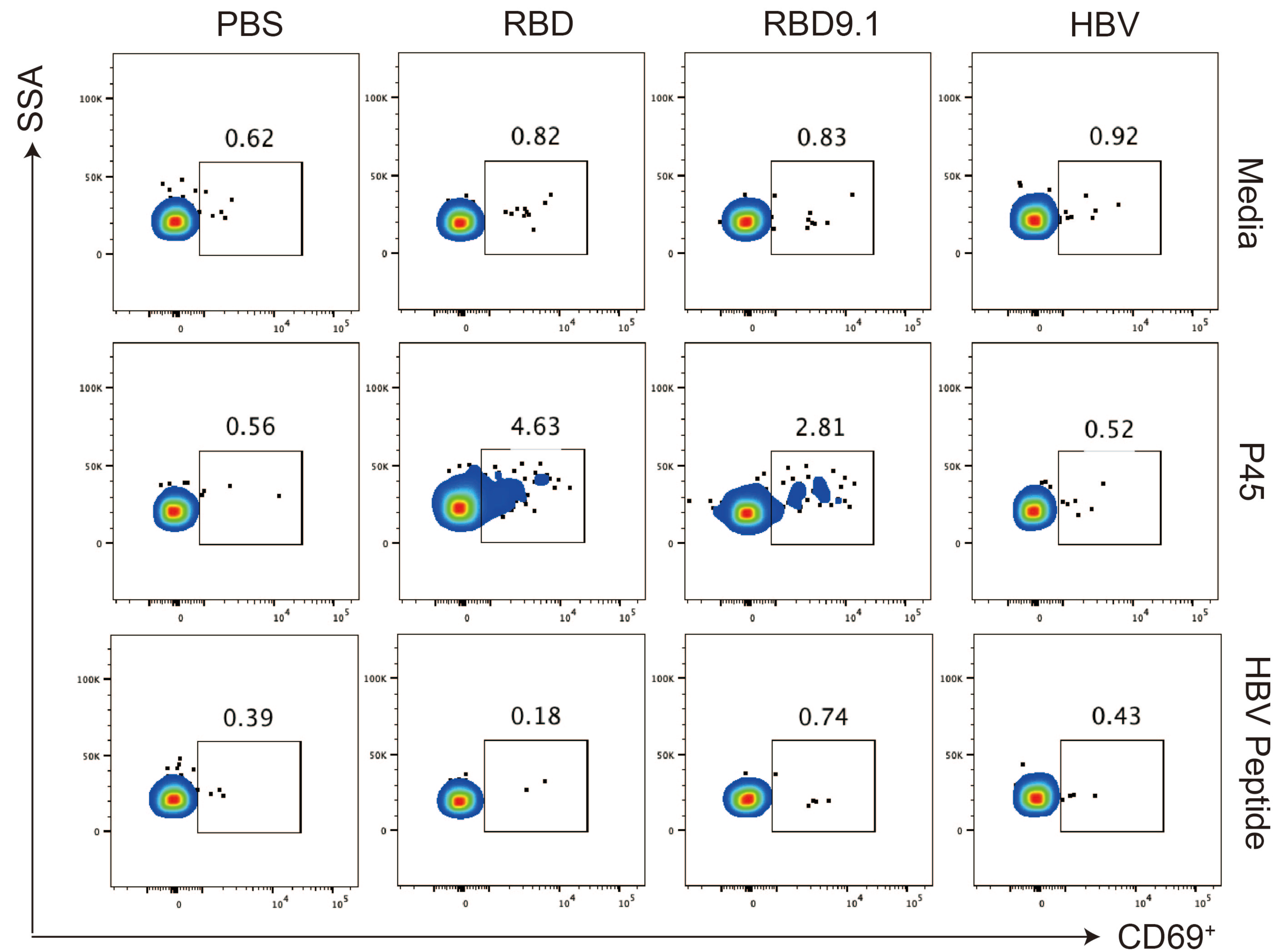

B.

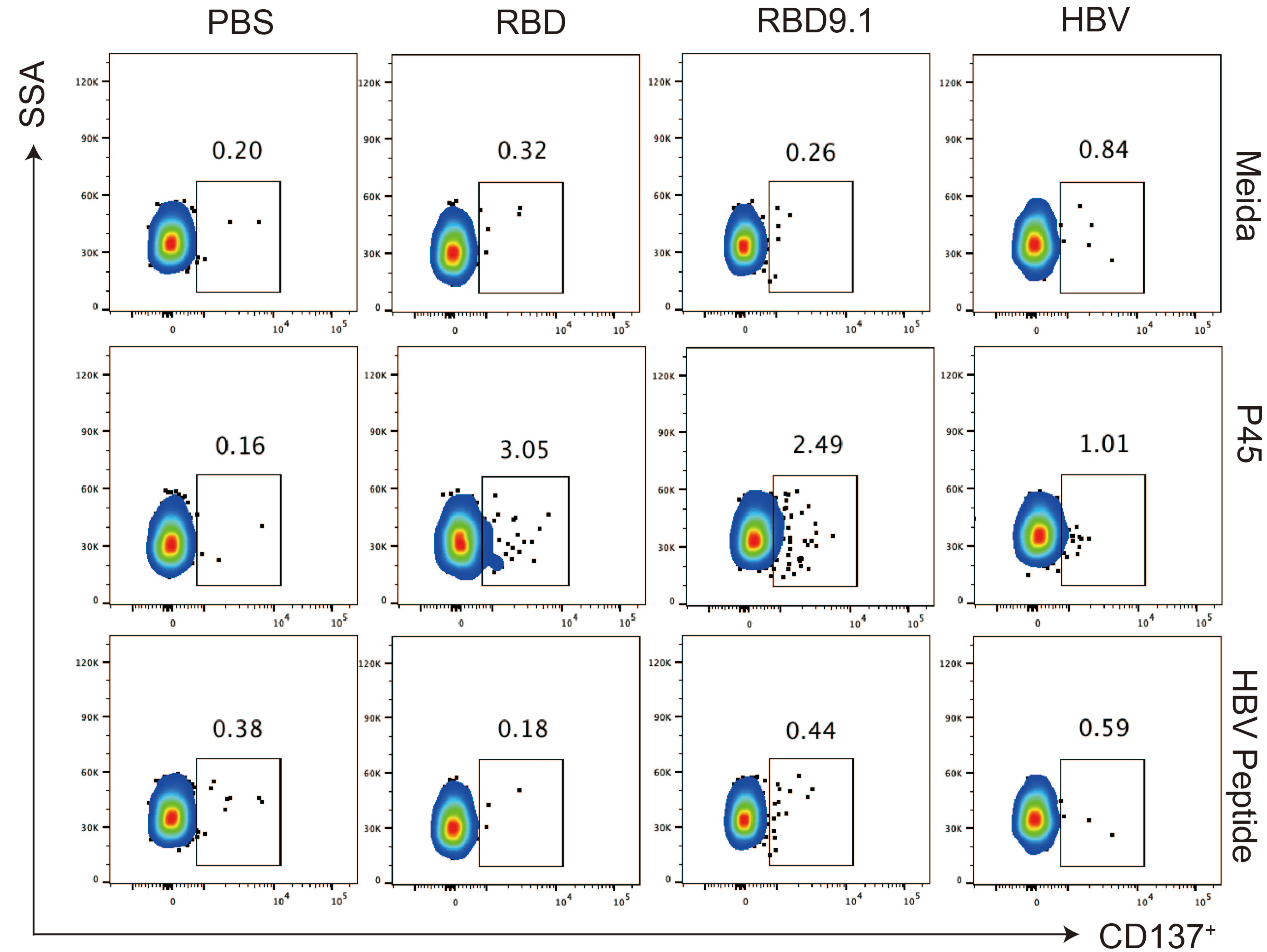

Supplement: Supplementary Figure 5 — Related to Figure 4 . The expression of CD137 (A) and CD69 (B) (gated on CD8+ T cells) after 10 μg/mL P45 or HBV peptide stimulation for 24 hours, normal media was used as negative control. [file Image_5.pdf]

Supplementary Figure.6

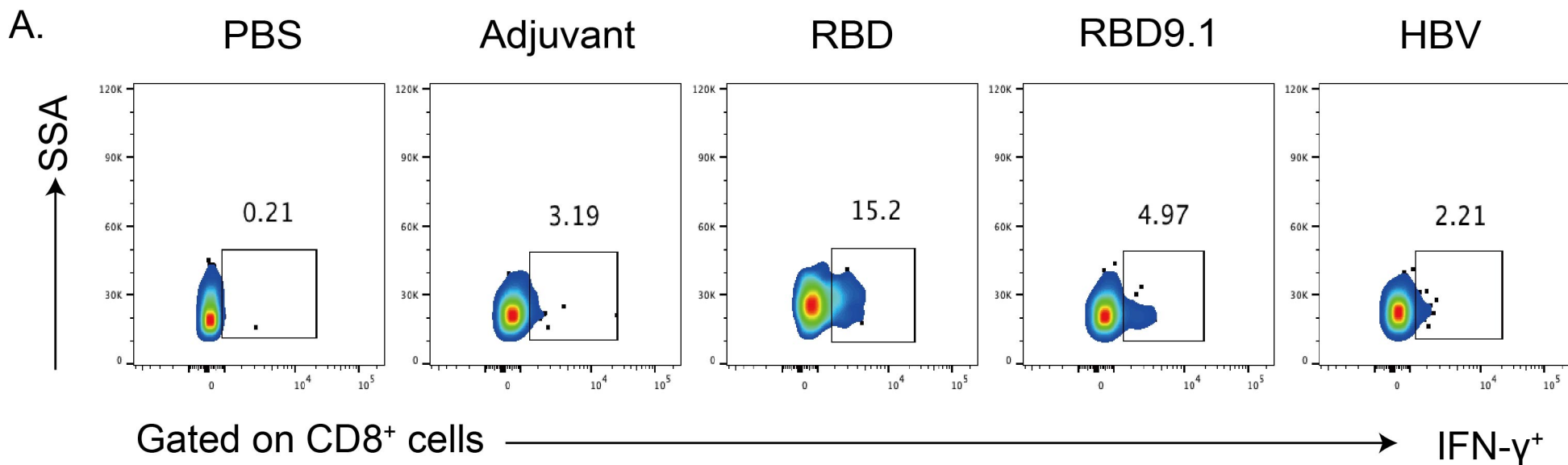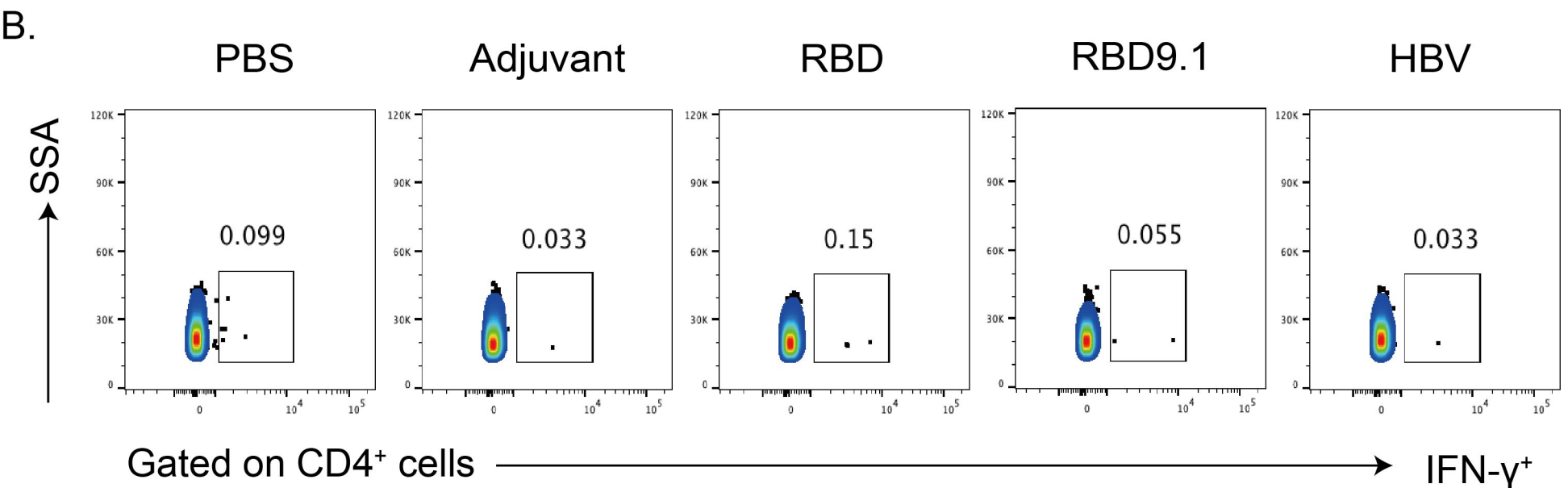

Supplement: Supplementary Figure 6 — Related to Figure 4 . The expression of IFN-γ gated on CD8+ T cells (A) and on CD4+ T cells (B) on the 10th day after the last immunization. [file Image_6.pdf]

A.

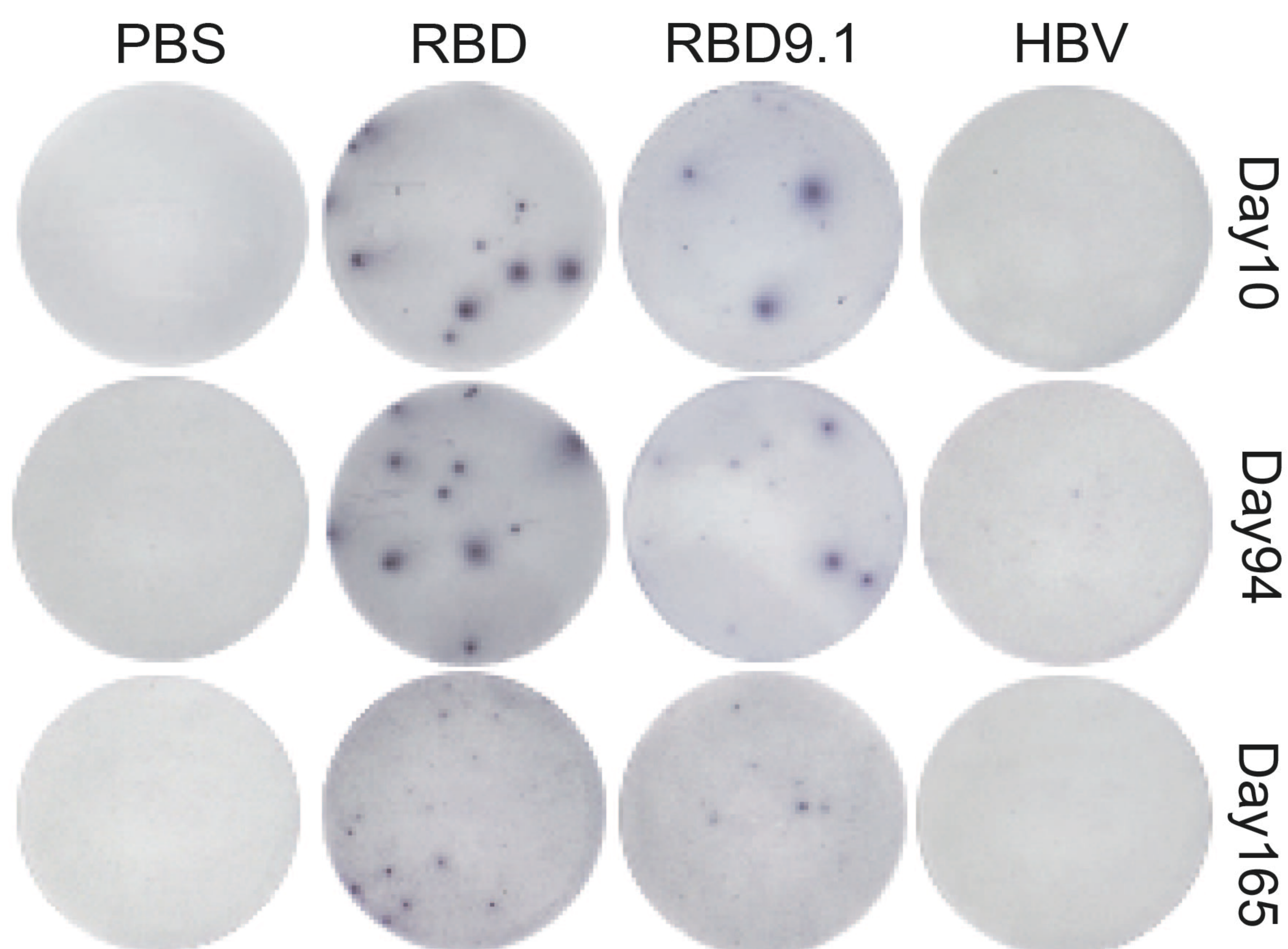

B.

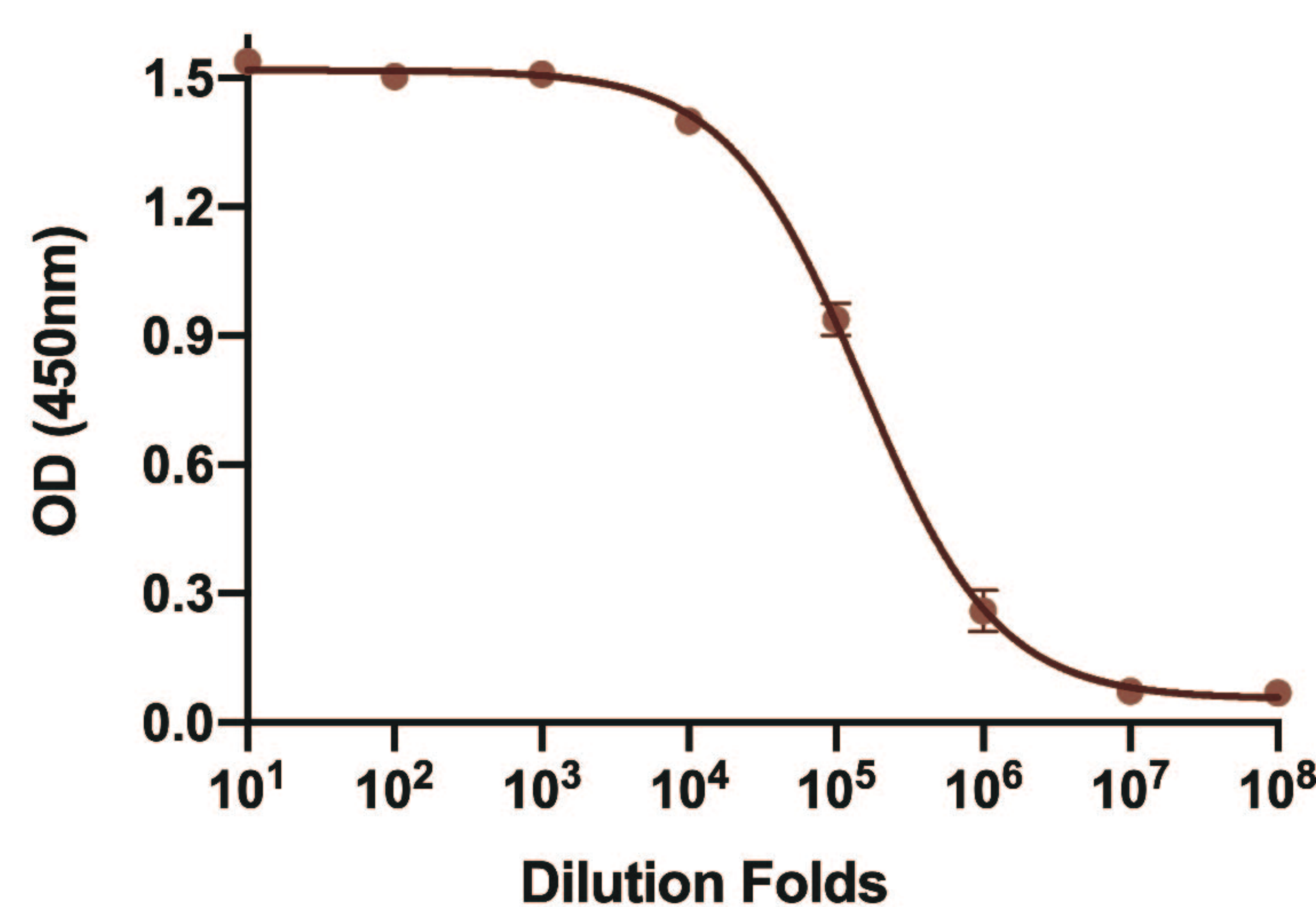

C.

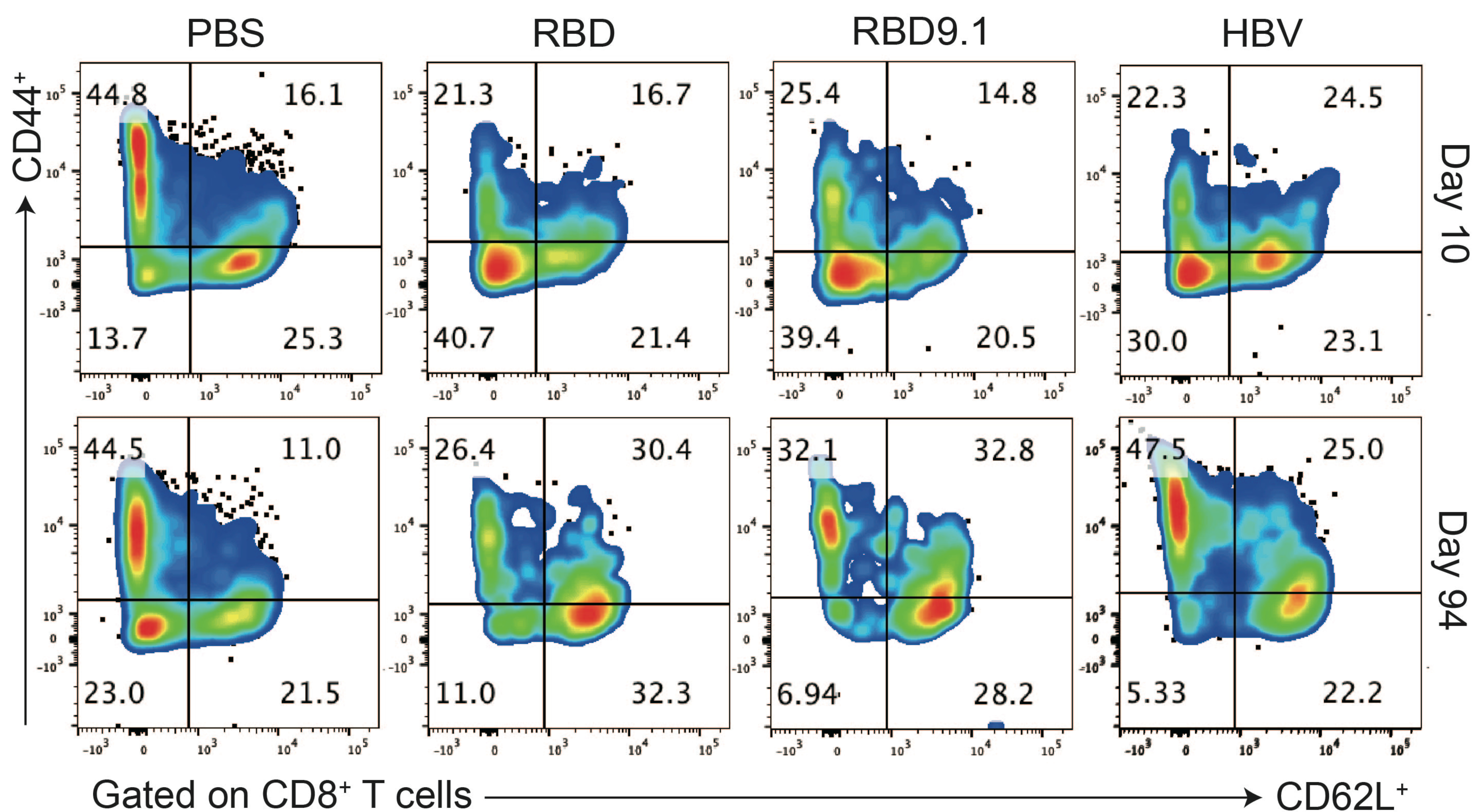

D.

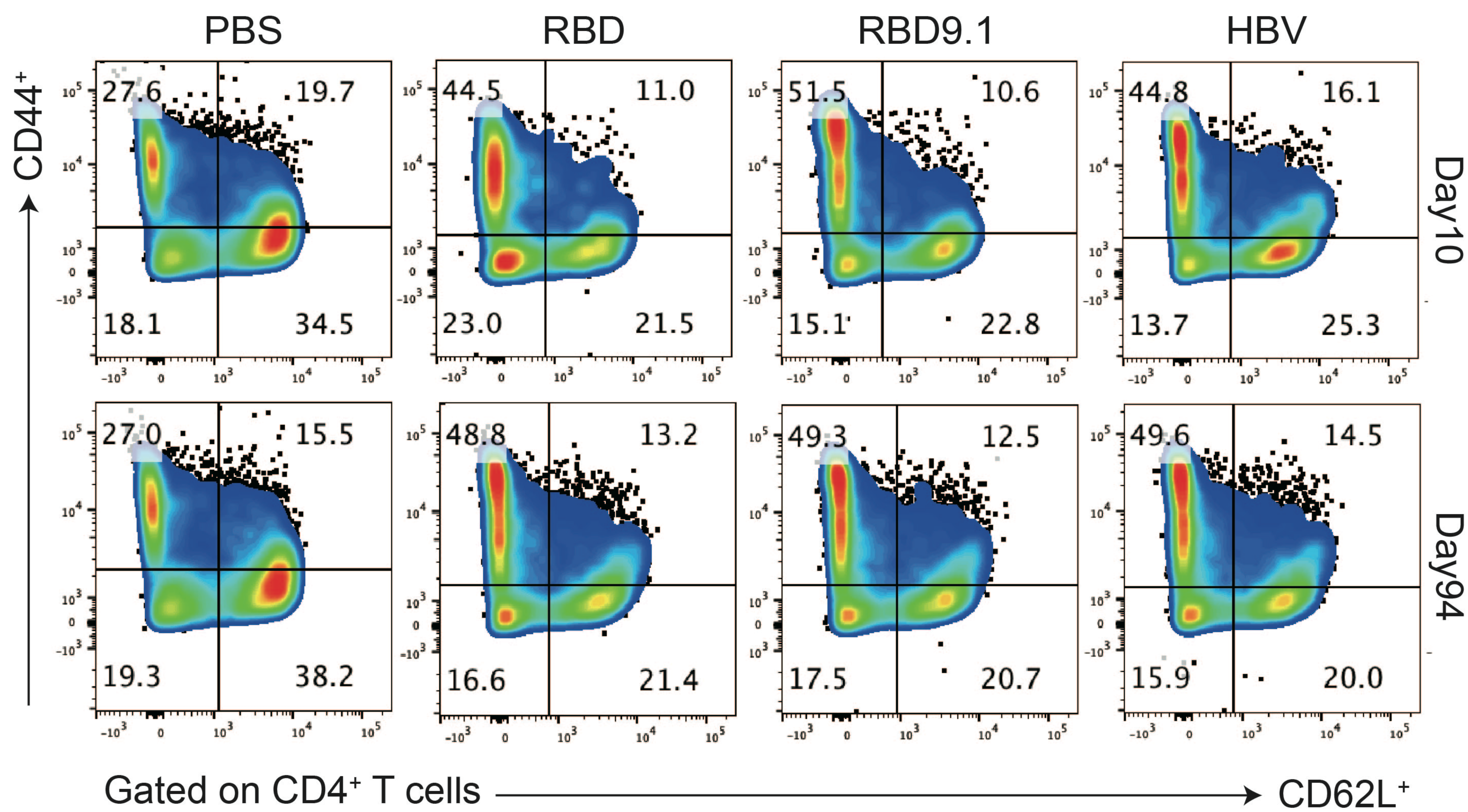

Supplement: Supplementary Figure 7 — Related to Figure 5 . (A) Stimulation with R848 and IL-2 for 6 days, the ELISPOT picture showed the number of RBD-specific IgG spots per 5 × 10^5^ splenocytes of each mouse. (B) The binding abilities of 10-fold serially diluted mouse serum to SARS-CoV-2 RBD recombinant protein. The ratio of Tn (CD62L+ CD44-), Te (CD62L- CD44-), Tem (CD62L- CD44+) and Tcm (CD62L+ CD44+) of CD8+ (C) or CD4+ T (D) cells on day 10 and day 94 after the last immunization. [file Image_7.pdf]
