# Supplementary material for: A Highly Conserved Peptide Vaccine Candidate Activates Both Humoral and Cellular Immunity Against SARS-CoV-2 Variant Strains
Source: Front Immunol. 2021 Dec 7;12:789905. doi: 10.3389/fimmu.2021.789905 (PMC8688401; doi:10.3389/fimmu.2021.789905)
Supplement: Supplementary Figure 2 — Immunization with RBD9.1 antigenic peptide efficiently induced neutralizing antibody production in Balb/c mice. (A) RBD9.1 and HBV peptide amino acid sequence information. (B) RBD9.1, RBD and HBV peptide immunization strategy. (C) SARS-CoV-2 RBD recombinant protein expressed in FreeStyle 293F cells and separated by affinity chromatography were verified by silver stain. (D) The production of HBV-specific antibodies from mice immunized with HBV peptide. (E) RBD9.1 sequence, with single point mutations (red) to alanine. (F) Sequence alignment for the RBD proteins of SARS-CoV-2 and SARS-CoV. The RBM region was marked in red, and the same amino acid sequence of the RBD protein of SARS-CoV-2 and SARS-CoV was marked in light blue. The amino acid sequence of RBD9.1 in SARS-CoV-2 RBD is marked with green dots. (G) Sequence alignment within the amino acid sequence of RBD9.1 of WT, P.1, B.1.1.7, B.1.351, B.1.617.1 and B.1.617.2 strains. [file Image_2.pdf]

Supplementary Figure 2

A.

Epitopes	Positions	Amino acide sequence
RBD9.1	444-463 (RBD)	KVGGNYNYLYRLFRKSNLKP
$S^{448-456}$ (P45)	448-456 (RBD)	NYNYLYRLF
HBV Peptide	14-32 (S1)	TNLSVPNPLGFFPDHQLDP

B.

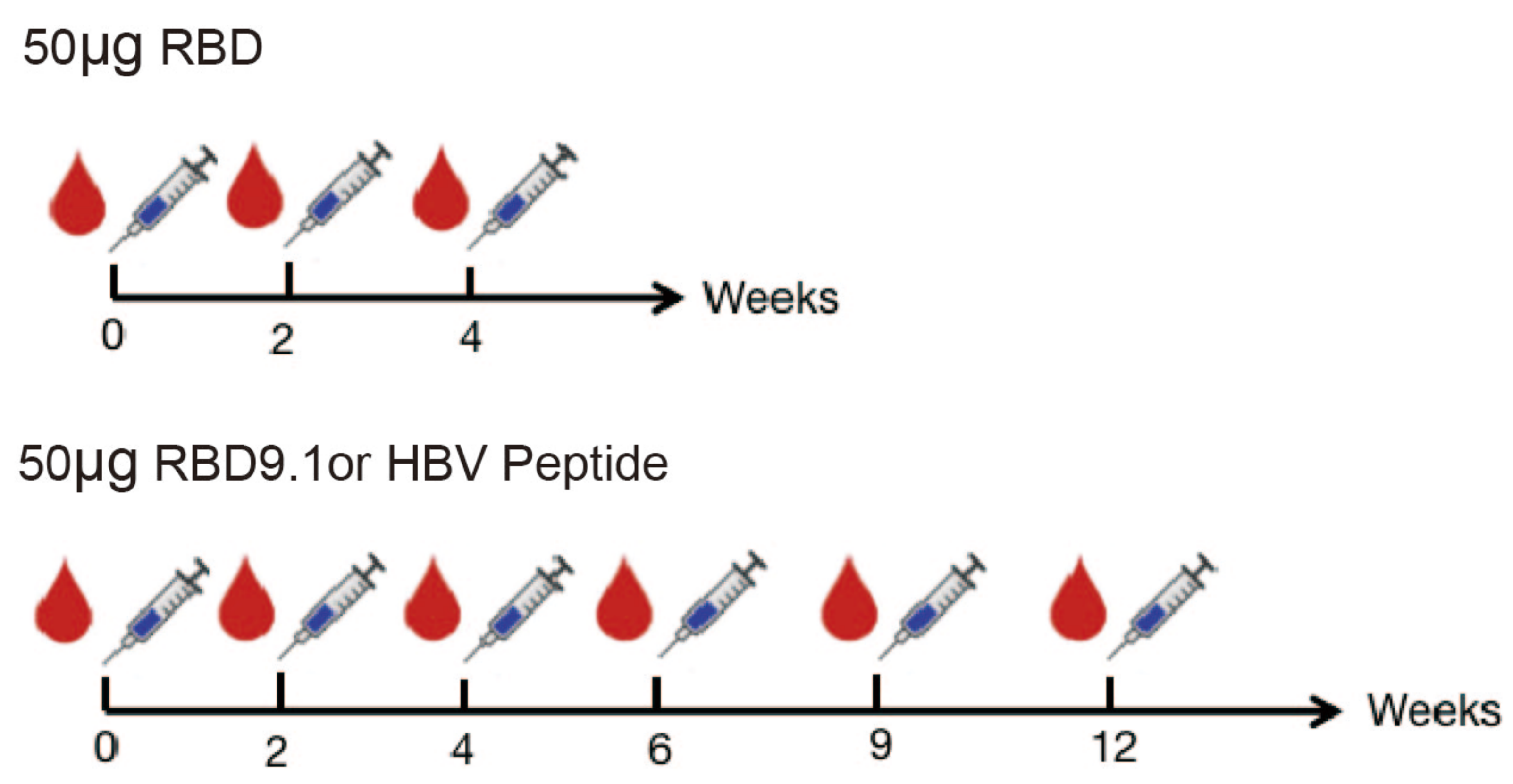

C.

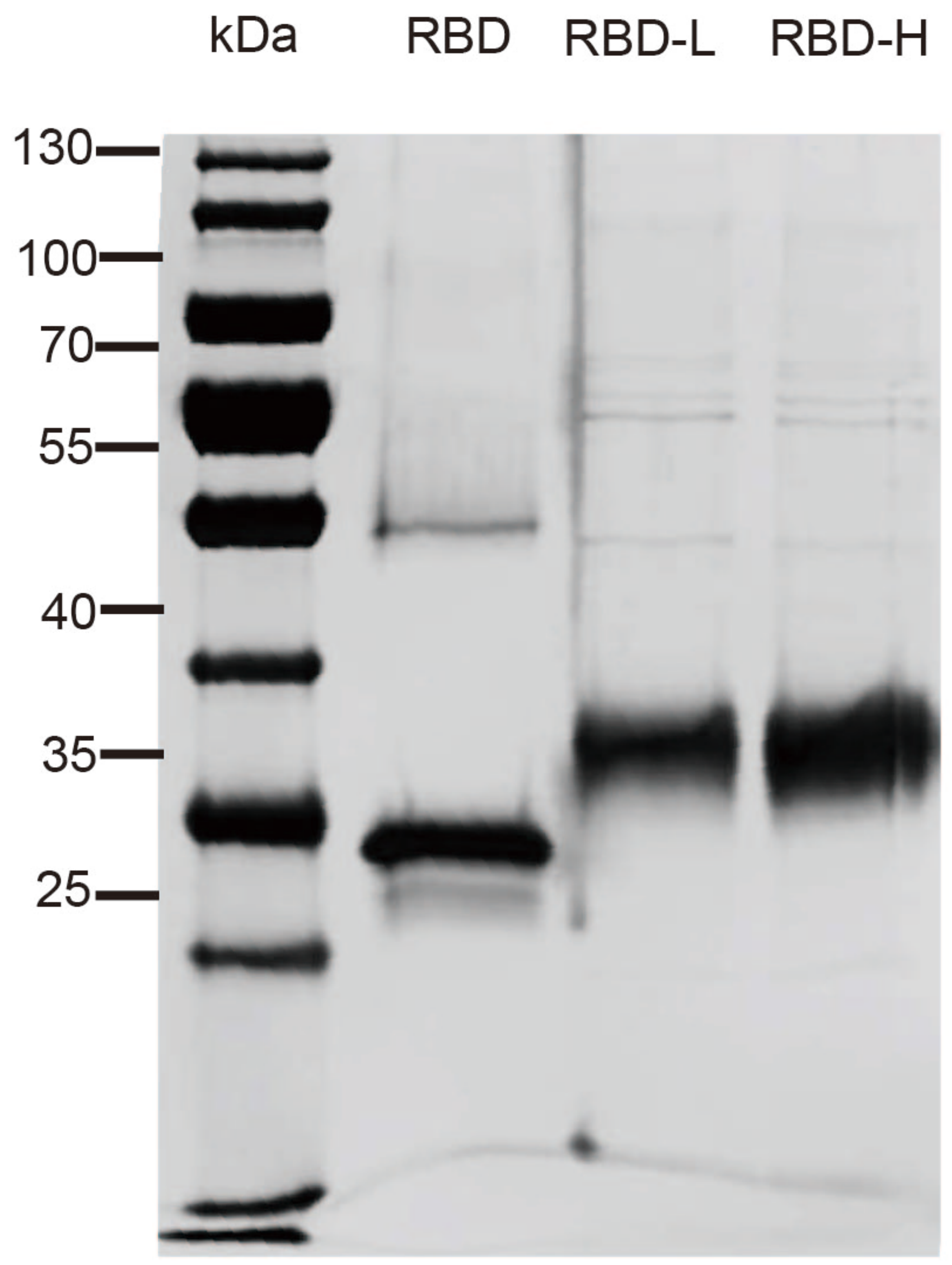

D.

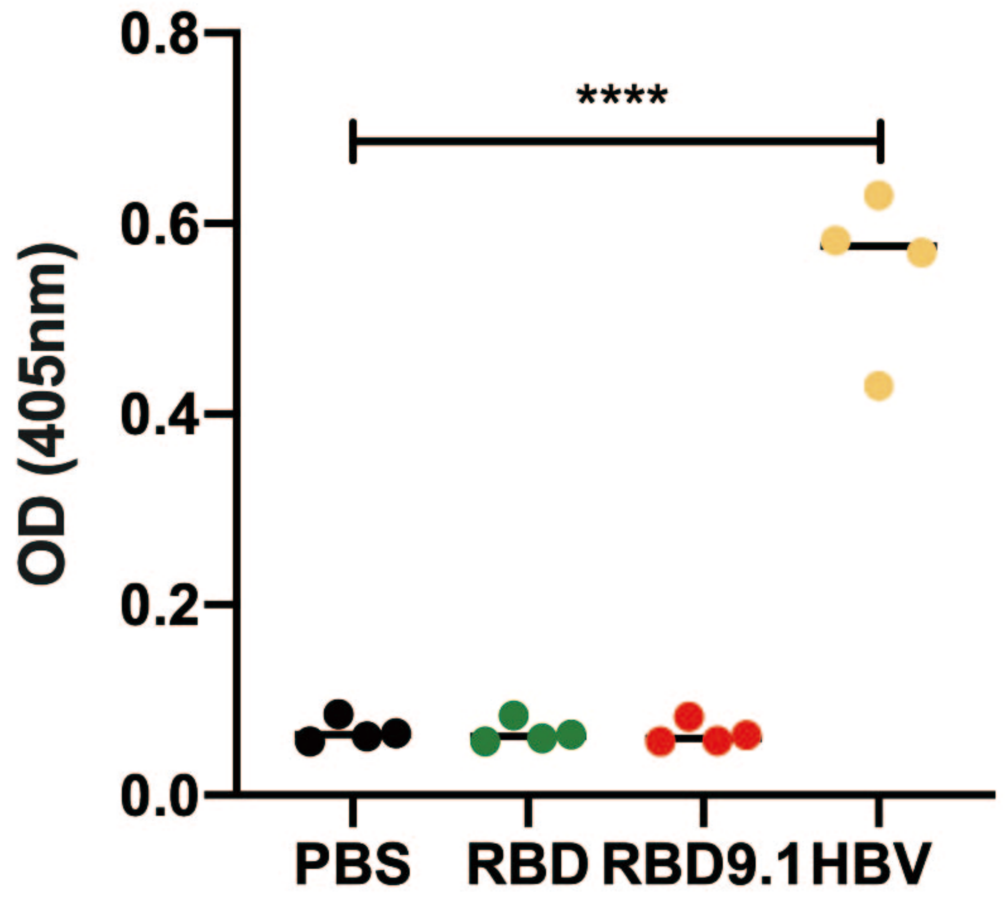

E.

Wildtype KVGGNYNYLYRLFRKSNLKP
K444A AVGGNYNYLYRLFRKSNLKP
V445A KAGGNYNYLYRLFRKSNLKP
G446A KVAGNYNYLYRLFRKSNLKP
G447A KVGANYNYLYRLFRKSNLKP
N448A KVGGAYNYLYRLFRKSNLKP
Y449A KVGGNANYLYRLFRKSNLKP
N450A KVGGNYAYLYRLFRKSNLKP
Y451A KVGGNYNALYRLFRKSNLKP
L452A KVGGNYNYAYRLFRKSNLKP
Y453A KVGGNYNYLARLFRKSNLKP
R454A KVGGNYNYLYALFRKSNLKP
L455A KVGGNYNYLYRAFRKSNLKP
F456A KVGGNYNYLYRLARKSNLKP
R457A KVGGNYNYLYRLFAKSNLKP
K458A KVGGNYNYLYRLFRASNLKP
S459A KVGGNYNYLYRLFRKANLKP
N460A KVGGNYNYLYRLFRKSALKP
L461A KVGGNYNYLYRLFRKSNAKP
K462A KVGGNYNYLYRLFRKSNLAP
P463A KVGGNYNYLYRLFRKSNLKA

F.

SARS-CoV-2 387LNDLCFTNVYADSFVI RGDEVRQIAPGQTGKIADYNYKLPDDFT GCV I AWN437
SARS-CoV 374LNDLCFSNVYADSFVVKGDDVRQIAPGQTGVIADYNYKLPDDFMGCVLAWN424

SARS-CoV-2 438SNNLDSKVGGNYNYLYRLFRKSN LKPFERDISTE IYQAGST PCNGVEGFNC488
SARS-CoV 425TRNI DATST GNYNYKYRYLRHGKLRPFERDISNVPFSPDGKPCT P- P ALNC474

SARS-CoV-2 489YF PLQSYGFQPTNGVGYQPYRVVVLSFE516
SARS-CoV 475YWPLNDYGFYTTT G I GYQPYRVVVLSFE502

G.

SARS-COV-2 Strains	The sequence of RBD9.1 in SARS-COV-2
WT	KVGGNYNYLYRLFRKSNLKP
P.1	KVGGNYNYLYRLFRKSNLKP
B.1.351	KVGGNYNYLYRLFRKSNLKP
B.1.1.7	KVGGNYNYLYRLFRKSNLKP
B.1.617.1	KVGGNYNYRYRLFRKSNLKP
B.1.617.2	KVGGNYNYRYRLFRKSNLKP
