# Supplementary material for: A Highly Conserved Peptide Vaccine Candidate Activates Both Humoral and Cellular Immunity Against SARS-CoV-2 Variant Strains
Source: Front Immunol. 2021 Dec 7;12:789905. doi: 10.3389/fimmu.2021.789905 (PMC8688401; doi:10.3389/fimmu.2021.789905)
Supplement: Supplementary Table 1 — The information of Groups and OD 405nm were listed in Supplementary Table 1 . And the threshold for grouping is 1.788, in accordance with the average OD value of 31 samples. [file Table_1.pdf]

	Patients ID	OD (405 nm)
Group 1	1	1.248
	9	1.506
	11	1.699
	12	1.678
	13	1.612
	14	1.719
	15	1.613
	17	1.18
	18	1.731
	19	1.708
	20	1.569
	21	1.788
	24	1.642
	25	1.278
	26	1.019
	27	1.246
	28	1.541
	29	1.455
	31	0.915
Group 2	2	2.186
	3	2.093
	4	1.91
	5	2.236
	6	2.262
	7	2.385
	8	2.098
	10	1.921
	16	1.989
	22	2.183
	23	2.247
	30	2.023
