# Supplementary material for: A Highly Conserved Peptide Vaccine Candidate Activates Both Humoral and Cellular Immunity Against SARS-CoV-2 Variant Strains
Source: Front Immunol. 2021 Dec 7;12:789905. doi: 10.3389/fimmu.2021.789905 (PMC8688401; doi:10.3389/fimmu.2021.789905)
Supplement: Supplementary Table 2 — The clinical information and RBD-hACE2 interaction inhibition titers (IC50) and SARS-CoV-2 pseudovirus neutralization titers (IC50) were listed in Table 2 . [file Table_2.pdf]

Table.2

Patient ID	Age(Years)	Gender(Male/Female)	Clinical classification (mild:0, severe:1)	ACE2-Competitive (IC_{50})	PsV NAb(IC_{50})
1	56	Female	0	--	--
2	45	Male	0	--	--
3	44	Male	0	--	--
4	38	Male	0	--	--
5	40	Female	0	27.69	1716
6	50	Male	0	43.14	3054
7	56	Female	0	233.5	--
8	51	Female	0	138	1556
9	48	Male	1	54.71	1640
10	32	Male	0	37.47	--
11	54	Male	0	--	--
12	43	Male	0	31.28	--
13	47	Female	0	26.96	2034
14	38	Male	0	--	199.2
15	33	Female	0	--	3358
16	44	Male	0	34.3	6878
17	60	Female	0	81.85	1441
18	48	Female	0	--	--
19	53	Female	0	24.49	739.7
20	57	Male	0	--	--
21	44	Male	0	55.4	1101
22	49	Male	0	4.6	--
23	23	Male	0	--	4648
24	44	Female	0	--	1311
25	59	Female	0	48.53	1619
26	20	Female	0	--	1309
27	37	Male	0	32.2	--
28	44	Female	0	--	1556
29	41	Male	0	43.92	--
30	66	Male	0	156.4	--
31	67	Female	0	30.64	--
