# Supplementary material for: A Highly Conserved Peptide Vaccine Candidate Activates Both Humoral and Cellular Immunity Against SARS-CoV-2 Variant Strains
Source: Front Immunol. 2021 Dec 7;12:789905. doi: 10.3389/fimmu.2021.789905 (PMC8688401; doi:10.3389/fimmu.2021.789905)
Supplement: Supplementary Table 3 — The information of immunized mice was listed in Table 3 . [file Table_3.pdf]

Table.3

Groups	Age (Weeks)	Gender (Male/Female)	Number	Mouse strain
PBS	6	Female	10	Balb/c
Adjuvant	6	Female	8	Balb/c
RBD	6	Female	10	Balb/c
RBD9.1	6	Female	10	Balb/c
HBV Peptide	6	Female	10	Balb/c
