# Supplementary material for: A Highly Conserved Peptide Vaccine Candidate Activates Both Humoral and Cellular Immunity Against SARS-CoV-2 Variant Strains
Source: Front Immunol. 2021 Dec 7;12:789905. doi: 10.3389/fimmu.2021.789905 (PMC8688401; doi:10.3389/fimmu.2021.789905)
Supplement: Supplementary Table 4 — The sequence information of SARS-COV-2 strains during RBD9.1 area were listed in Table 4 . [file Table_4.pdf]

Table.4

SARS-CoV-2 Strains	The sequence of RBD9.1 peptide
WT	KVGGNYNYLYRLFRKSNLKP
P.1	KVGGNYNYLYRLFRKSNLKP
B.1.351	KVGGNYNYLYRLFRKSNLKP
B.1.1.7	KVGGNYNYLYRLFRKSNLKP
B.1.617.1	KVGGNYNYRYRLFRKSNLKP
B.1.617.2	KVGGNYNYRYRLFRKSNLKP
