# Supplementary material for: A Highly Conserved Peptide Vaccine Candidate Activates Both Humoral and Cellular Immunity Against SARS-CoV-2 Variant Strains
Source: Front Immunol. 2021 Dec 7;12:789905. doi: 10.3389/fimmu.2021.789905 (PMC8688401; doi:10.3389/fimmu.2021.789905)

Supplementary Figure. 8

A.

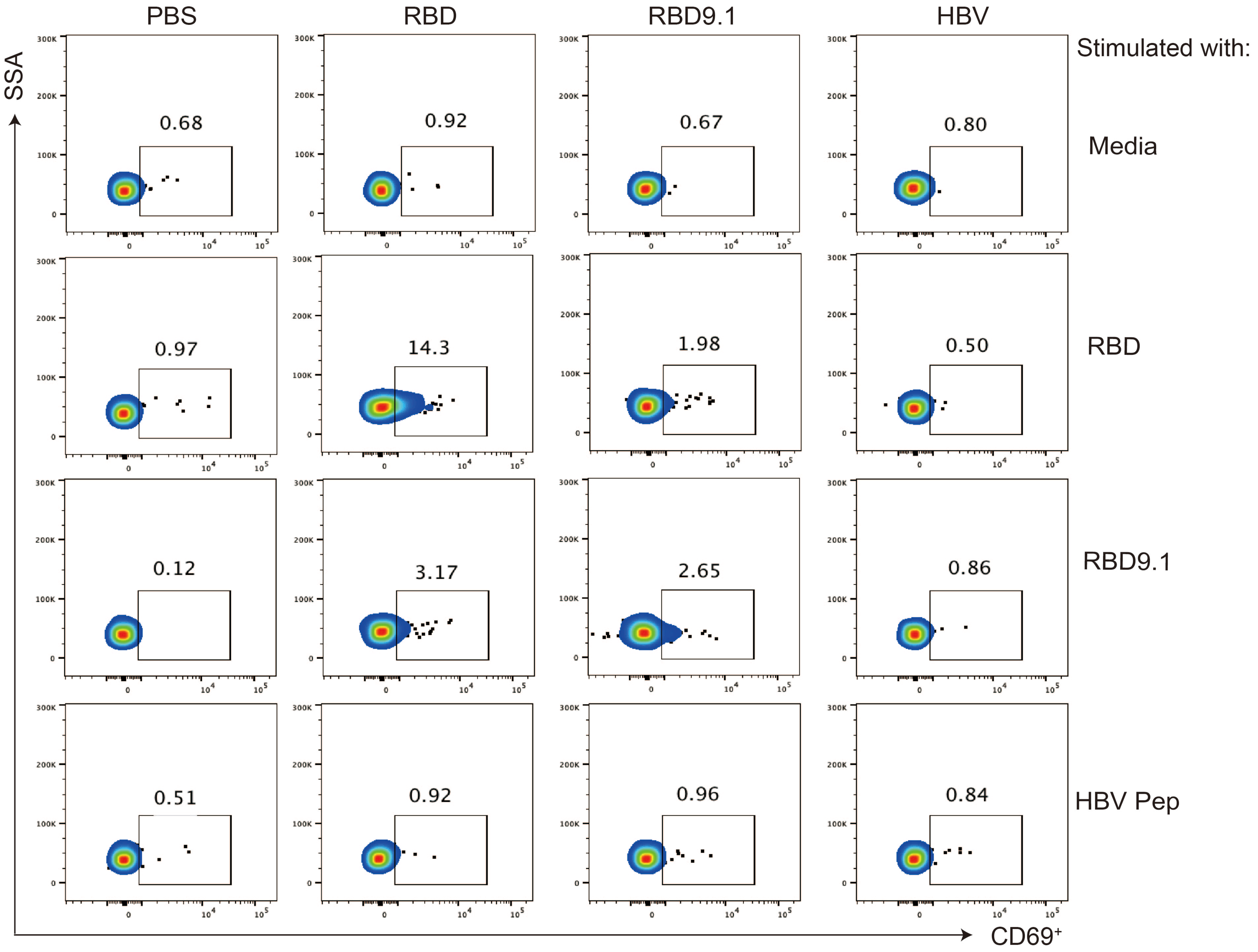

B.

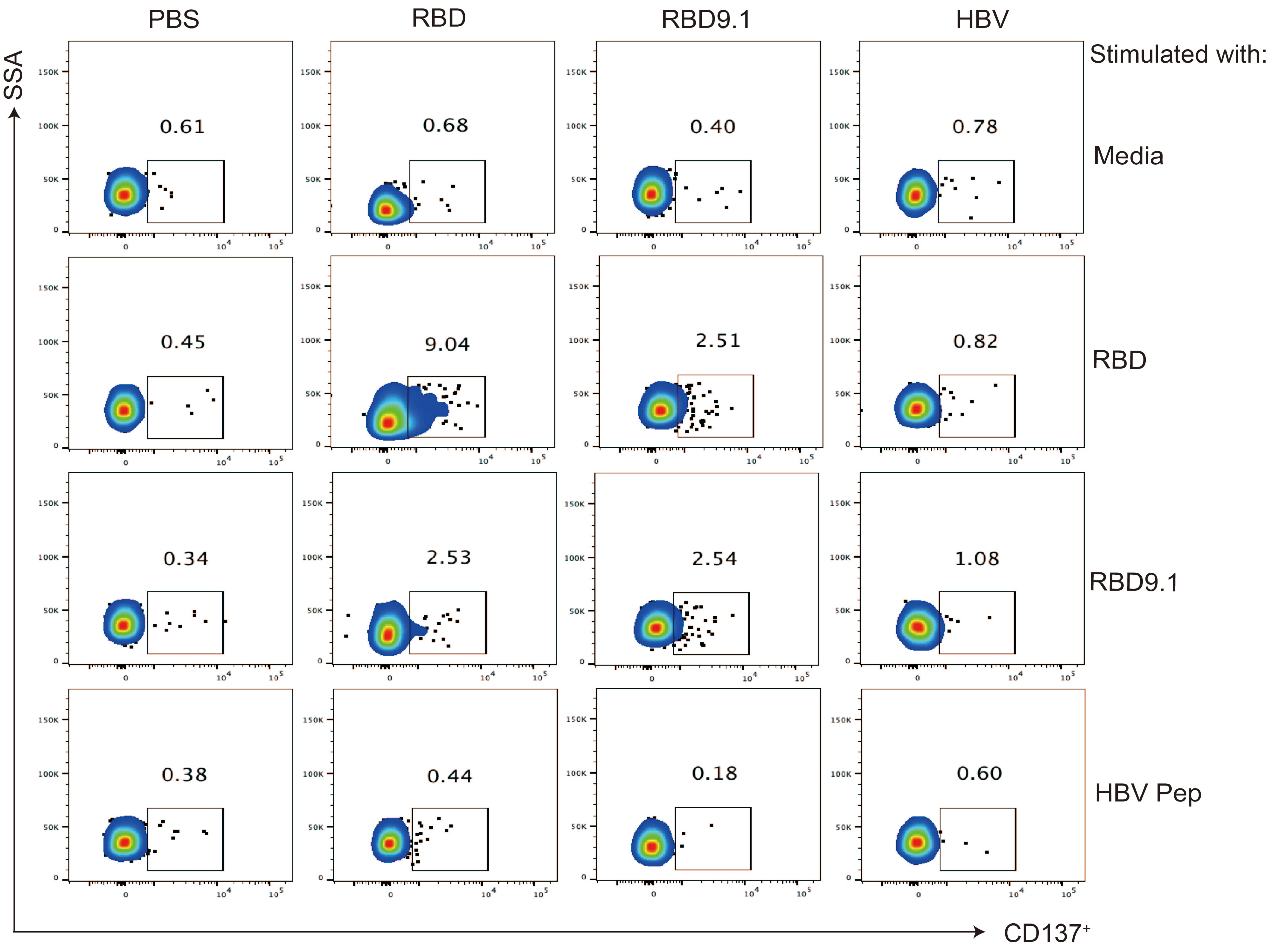

Supplement: Supplementary Figure 8 — Related to Figure 6 . The expression of CD69 (A) and CD137 (B) (gated on CD8+ T cells of immune mice) were detected by flow cytometry after 10 μg/mL RBD, RBD9.1 or HBV peptide stimulation for 24 hours, normal media was the negative control. [file Image_8.pdf]
